# A phylogeny for genus *Capra* based on extensive sampling of wild populations

**DOI:** 10.1371/journal.pone.0334624

**Published:** 2025-10-27

**Authors:** Steve Jordan, Saeid Naderi, Hamid-Reza Rezaei, Gordon Luikart

**Affiliations:** 1 Department of Biology, Bucknell University, Lewisburg, Pennsylvania, United States of America; 2 Laboratoire d’Ecologie Alpine, CNRS UMR 5553, Universite ˊGrenoble Alpes, Grenoble, France; 3 Environmental Sciences Department, Faculty of Natural Resources, University of Guilan, Rasht, Iran; 4 Environmental Sciences Department, Faculty of Fisheries and Environmental Sciences, Gorgan University of Agricultural Sciences and Natural Resources, Gorgan, Iran; 5 Flathead Lake Biological Station, Montana Conservation Genomics Laboratory, University of Montana, Polson, Montana, United States of America; Central University of Kerala, INDIA

## Abstract

Among the most economically and ecologically important taxa are those with wild and domestic counterparts, such as the true goats (genus *Capra*), that are distributed and used by humans world-wide. Such taxa often played roles in the spread of pastoralism, farming, and modern societies. To advance understanding of the *Capra*, a relatively recent radiation across Eurasia, we generated one of the most complete taxonomic datasets for this genus to date. It includes 4603 bp of DNA sequence data for 11 nuclear loci from a broad geographic distribution of *Capra*, including 52 wild and 10 domestic individuals from nine species and 21 countries. All wild species were sampled in the wild (not in captivity). Results support the monophyly of recognized IUCN (International Union for Conservation of Nature) taxa, *C. ibex*, *C. nubiana*, *C. pyrenaica*, and *C*. *sibirica* while offering little support for the monophyly of *C. aegagrus*, *C*. *falconeri*, *C. hircus*, *C. caucasica*, or *C. cylindricornis*. We confirm wild goats (*C. aegagrus*) are the likely sole ancestor of domestic goats. This study bolsters and confirms prior studies, demonstrating the usefulness of multiple independent nuclear genes and widespread sampling of wild and domestic individuals for studies of taxa important to humans.

## Introduction

Understanding the origins, spread, and relationships among human-allied taxa, such as domestic and wild goats, is important to the study of human societies and economies, as well as efforts to untangle complex ecological and evolutionary processes [1]. The ungulate genus *Capra* includes cosmopolitan domestic goats and wild goat species (bezoars, ibex, markhors, and turs) with natural distributions throughout mountainous habitats in the southern Palearctic in Europe, Africa, and Asia, from northern Mongolia and Russia to western Europe and Ethiopia. It is likely that goats were among the first domesticated ungulates, and that *C. aegagrus* is the ancestor of all domestic goats [2,3]. Fossil data suggest that the *Capra* ancestral lineage originated perhaps as early as middle Miocene (8.7–11.9 YBP), with a species radiation occurring across a broad geographical area during the Plio-Pleistocene [4,5]. Moreover, it is clear that wild species can hybridize freely in captivity, and can produce fertile offspring [6].

In a prior study [7], we reviewed the molecular, morphological, and behavioral evidence that has been brought to bear on the taxonomy and evolutionary history of this genus. Early workers principally determined species boundaries and relationships using horn morphology and color, characters that now appear labile, and that have likely been influenced by convergent evolution [8].

It is critically important that studies of charismatic taxa that are prized by hunters and zoos alike include thorough sampling of wild populations, which can be extremely difficult due to their widespread geographic distributions and the steep, remote terrain they often inhabit. Captive populations are easy to sample, but come with many potential phylogenetic problems. For example, they can include individuals resulting from unknown, complicated processes, like hybrids of known species, hybrids or back crosses between non-naturally co-occurring populations, individuals descending from severely bottlenecked or inbred populations, or individuals from populations that have been artificially selected for certain attributes or not allowed to experience natural selection. All of these circumstances can create patterns of genetic divergence that are not representative of wild evolutionary and ecological processes.

Pioneering molecular studies of *Capra* included data from captive animals and incomplete taxonomic, geographic, and/or genomic sampling [7,9–14]. Recent studies have vastly improved genomic and geographic sampling [15–17], including genomic sequencing or genome-wide SNP analysis. The most thorough recent dataset is found in Pogorevc et al. [17], and includes widespread geographic and taxonomic sampling. However, some DNA samples in that study were taken from captive animals. Therefore, the phylogenetic literature on the genus *Capra* would benefit from a broad taxonomic, geographical, and molecular study of wild populations. Here we present data from 11 nuclear genes from 52 wild *Capra* representing all known, extant species except the Walia ibex, and many populations within them, in an effort to better understand the evolutionary history of this important genus.

## Materials and methods

### Taxon sampling

**Fig 1:**
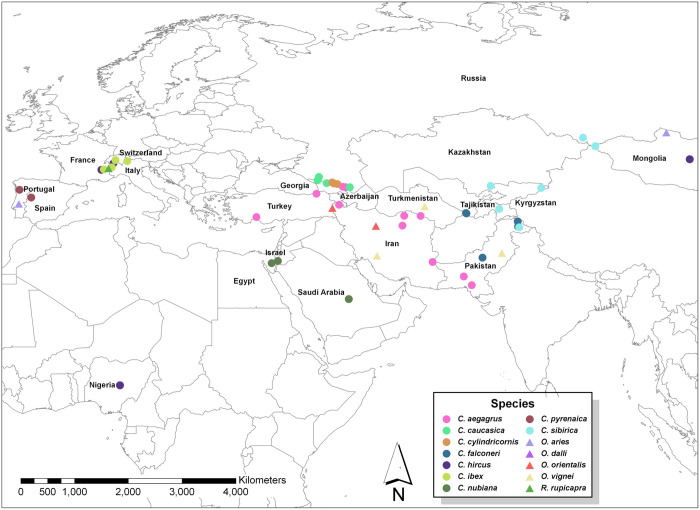
Geographic origins of our 71 samples from the 14 species, including all 9 *Capra* species.

Guided by the taxonomy currently accepted by IUCN [18], we obtained tissue samples from 62 wild and domestic *Capra* individuals representing nine species and 21 countries (Table 1, Fig 1). Of these, 52 individuals are from wild species, with tissue collected in the wild in 20 countries. Tissue samples included skin, horn, and fecal materials collected by us or by collaborators. Most of the material came from hunter-killed individuals sampled by in-country managers, and species identities were confirmed in the field by experienced *Capra* biologists. We also included nine outgroup individuals from available species of *Ovis* (sheep) and one *Rupicapra* (chamois), which are both closely related to *Capra*.

**Table 1:** Individuals sampled and their geographic locations of origin from wild populations (none from zoos).

Specimen Number	Species	Sampling Location
CaIRI277A	*Capra aegagrus*	Sistan Province, Iran
CaAk2	*C. aegagrus*	Antalya Province, Turkey
CaAz5	*C. aegagrus*	Nakhchivan Autonomous Republic, Azerbaijan
CaPaDurP10	*C. aegagrus*	Baluchistan Region, Pakistan
Cach104	*C. aegagrus*	Baluchistan Region, Pakistan
CaDak40	*C. aegagrus*	Republic of Daghestan, Russia
CaDak63	*C. aegagrus*	Republic of Daghestan, Russia
CcaD62	*C. caucasica*	Republic of Karachay-Cherkess, Russia
CcaSz9	*C. caucasica*	Svanetia Province, Georgia
CcaD14	*C. caucasica*	Republic of Daghestan, Russia
CcaTt47	*C. caucasica*	Karachay-Cherkess Republic, Russia
CcyRusD45	*C. cylindricornis*	Republic of North Ossetia-Alania, Russia
CcAz23	*C. cylindricornis*	Azerbaijan
CcAs4	*C. cylindricornis*	Mtskheta-Mtianeti Region, Georgia
CcDNO440	*C. cylindricornis*	Republic of Daghestan, Russia
CcYs452	*C. cylindricornis*	Republic of North Ossetia-Alania, Russia
CfTk375	*C. falconeri*	Lebap Province, Koytendag District, Turkmenistan
CfUk1	*C. falconeri*	Surkhandarya Province, Uzbekistan
CfPaTor02	*C. falconeri*	Torghar District, Pakistan
CfPaTor05	*C. falconeri*	Torghar District, Pakistan
CfPk14m	*C. falconeri*	Ziarat District, Balochistan, Pakistan
CfTk616	*C. falconeri*	Lebap Province, Koytendag District, Turkmenistan
CfF1	*C. falconeri*	Gilgit-Baltistan, Pakistan
ChFrAlp16	*C. hircus*	Rhône-Alpes, France
ChFrAlp07	*C. hircus*	Rhône-Alpes, France
ChSa133	*C. hircus*	Switzerland
ChMo39	*C. hircus*	Mongolia
ChMo57	*C. hircus*	Mongolia
ChMo76	*C. hircus*	Mongolia
ChMo82	*C. hircus*	Mongolia
Chwe73	*C. hircus*	Nigeria
Chwe79	*C. hircus*	Nigeria
Chwe99	*C. hircus*	Nigeria
Cii8	*C. ibex*	Rhône-Alpes, France
CiGP14.1V	*C. ibex*	Aosta Valley, Italy
Ci67.98	*C. ibex*	Grisons Canton, Switzerland
CiAu24499	*C. ibex*	Bern Canton, Switzerland
CiGP1	*C. ibex*	Aosta Valley, Italy
CiNES20	*C. nubiana*	South Sinai, Egypt
CiNi19	*C. nubiana*	Israel
CiNiSB8	*C. nubiana*	Israel
CiNiY27	*C. nubiana*	Israel
CiNH1	*C. nubiana*	Hawtat Bani Tamim, Riyadh Region, Saudi Arabia
CiNH2	*C. nubiana*	Hawtat Bani Tamim, Riyadh Region, Saudi Arabia
CiNH3	*C. nubiana*	Hawtat Bani Tamim, Riyadh Region, Saudi Arabia
CiNH4	*C. nubiana*	Hawtat Bani Tamim, Riyadh Region, Saudi Arabia
CinH562	*C. nubiana*	Hawtat Bani Tamim, Riyadh Region, Saudi Arabia
Cpyr6	*C. pyrenaica*	Spain
CAB14	*C. pyrenaica*	Portugal
C.pyr257	*C. pyrenaica*	Salamanca Province, Las Batuecas, Spain
CisTajKa488	*C. sibirica*	Gorno-Badakhshan, Pamir Mountains, Tajikistan
CisTajT460	*C. sibirica*	Gorno-Badakhshan, Pamir Mountains, Tajikistan
CisaKir1	*C. sibirica*	Kyrgyzstan
CisfKaz1	*C. sibirica*	Kazakhstan
CisRus22	*C. sibirica*	Altai Republic, Russia
CiSP454	*C. sibirica*	Gilgit-Baltistan, Pakistan
CisM10	*C. sibirica*	Khovd Province, Mongolia
CisM18	*C. sibirica*	Ömnögovi Province, Mongolia
CisM57	*C. sibirica*	Ömnögovi Province, Mongolia
CisM58	*C. sibirica*	Ömnögovi Province, Mongolia
CisM87	*C. sibirica*	Övörkhangai Province, Mongolia
CisM9	*C. sibirica*	Ömnögovi Province, Mongolia
OarMH1	*Ovis aries*	Khövsgöl Province, Mongolia
902MB	*O. aries*	Portugal
OvDaBC002	*O. dalli*	British Columbia, Canada
OoI1001	*O. orientalis*	Tehran Province, Iran
OggTK1	*O. orientalis*	Van Province, Turkey
OvIRI168	*O. vignei*	Southwest Iran
OvPak108	*O. vignei*	Pakistan
OvaTkm14	*O. vignei*	Turkmenistan
Rr573800	*Rupicapra rupicapra*	France

### Primer design

We generated DNA sequence data for 11 nuclear loci selected in anticipation of appropriate variation for phylogenetic questions. These loci are zona pellucida 2 (ZP2), zona pellucida 3 (ZP3), growth differentiation factor 9b (GDF9B), kappa casein (KapCas), keratin associated protein 1.3 (KAP1.3), toll-like receptor 2 (TLR2), toll-like receptor 4 (TLR4), non-coding regions flanking an anonymous microsatellite locus (U80588), interleukin 4 (IL4), interleukin 16 (IL16), and CWC15 spliceosome associated protein homolog (CWC15). Details on these loci including length sequenced, primer sequences, and optimal maximum likelihood model can be found in Tables 2 and 3.

**Table 2:** Attributes of each locus, including length, the best model of evolution, and variability by site category.

Locus	BP	Subst. Model	No. variable sites Ingroup		All taxa	
			Var	PI	Var	PI
GDF9B	481	JC	5	1	11	4
CWC15	296	GTR	6	6	10	7
IL4	451	GTR + I	9	6	19	11
IL16	432	K80	11	6	26	10
Kap1.3	583	HKY + I + G	15	8	23	11
KapCas	493	HKY + I	16	9	34	15
TLR2	468	GTR	4	2	15	7
TLR4	375	HKY + I	6	4	15	8
U80588	239	GTR + I	10	6	14	10
ZP2	310	SYM + I	11	7	15	7
ZP3	475	GTR + I	29	13	41	18
All loci	4603	GTR + I + G	122	68	223	108

BP = length in base pairs; Var = number of variable sites; PI = number of parsimony informative sites.

**Table 3:** Primers and PCR conditions for each included locus.

Locus	Tm	F name	F Primer	R Name	R Primer
GDF9B	58	GDF9BF	ACTCCGCTTCGTGTGTCAGC	GDF9BR	TACTCCCATTTGCCTCAATC
CWC15	56	CWC15148-F	GGGATGATGACGTTGTTTTC	CWC15148-R	GGGTTAAACCAATTCCCAAG
IL4	55	IL4-X1F	TCACATTGTCAGTGCAAATAGAG	IL4-X2R	TTTGGGGCAGCAAAGACGT
IL16	58	IL16-F	CCAGGCAAGCTGTGATCGT	IL16-R	GAAGATCCTGTTAACTGTCAGAGG
Kap1.3	58	KAP1-3F	GGGTGGAACAAGCAGACCAAACTC	KAP1-3R	AAGTTTGTTGGGACTGTACACTGGC
KapCas	50	Kcas-X4F	AGAAATAATACCATTCTGCAT	Kcas-X4R	GTTGTCTTCTTTGATGTCTCCTTAGAG
TLR2	58	TLR2-X2fa	GACCTGCAGAGGTGTGTGAA	TLR2-X2ra	TGAAAAATGGAAAGTGTGCAA
TLR4	58	TLR4-X4fb	TTCAAGGGTTGCTGTTCTCA	TLR4-X4rb	CAGCACCTGAAGGCTAGAGAG
U80588	58	U80588-Fb	AGTATCTTTTCTTGCATTTGTTTCC	U80588-Rb	CACAGGGGTTTCTGGTTGG
ZP2	58	ZP2-X8F	CCATCTCTACATGGTGCCTCT	ZP2-X9R	TTGTTTTGAGGAGAGTTTTGCT
ZP3	58	ZP3A-X3F	TGCCATTCAGGACCACAGT	ZP3A-X4R	GGAAGTCCACGATGGTGTG
ZP3	58	ZP3A-X4F	GAGAAGATGACGCCCACCT	ZP3A-X5RA	CATTAGCAAAACGGAACACATC

Primers for most of these loci were designed using the protocol described by Jordan et al. [19]. In brief, we used the ICCARE [20] website (http://genoweb.toulouse.inra.fr/iccare/) to align ESTs from *Bos taurus* with human genome sequences. We located primers in exon regions that were largely conserved between these taxa so that PCR amplified regions spanning introns.

### DNA extraction PCR and sequencing

We extracted DNA using Qiagen DNEasy tissue kits. High-throughput PCR and DNA sequencing were performed using standard protocols. We used the AmpliTaq gold DNA polymerase (ABI). This is a hot start molecule requiring that each PCR begin with 10 min at 95°C. We then ran 35 cycles consisting of 95°C for 30 s, annealing temperatures of 50–58°C (Table 3) for 30–60 s, and 72°C for 60 s. PCR purification and sequencing of both DNA strands was carried out by Genome Express (Meylan, France) using an ABI 3730 sequencer (Applied Biosystems) using the POP 7 polymer and standard conditions.

### Sequence alignment, model fitting and data exploration

DNA sequences were assembled and aligned using SeqScape v2.6 (ABI), Aligner (CodonCode Corp.), and MEGA [21] and adjusted by eye.

We selected appropriate maximum likelihood (ML) models using MrModelTest [22]. We developed three data partitioning schemes and selected appropriate models for each (11-, four-, and one-model analyses, see below). We also ran neighbor joining (NJ) analyses for each locus individually including 1) the ingroup only and 2) both the ingroup and outgroup, using ML distance settings corresponding to the GTR model. Parameter estimates from these analyses are included in S1 File.

### ML and Bayesian analysis

We ran a ML analysis using PAUP* [23], the GTR + I + G model, TBR branch swapping and 10 addition-sequence replicates. Initial model parameter values were estimated using a NJ tree and fixed for the first ML run (see S1 File for estimates). We ran a second, identical ML analysis with parameter values fixed to those estimated from the first ML run. We then ran 175 bootstrap (BP) replicates, using one addition-sequence replicate for each, and with parameter values fixed to those estimated from the second ML tree.

We carried out three Bayesian analyses using MrBayes [24] and the following model schemes: 1) mixed-models with a separate model for each locus (11 models), 2) mixed-models with separate models based on codon position (four models: 1^st^, 2^nd^, and 3^rd^ codon positions and noncoding regions), and 3) a single model for the entire alignment (one model). We ran each of these three analyses for 100 million generations using five chains (four heated, one cold) and used Tracer [25] to visually assess chain stationarity for burnin. The burnin was 25M generations for the 11-model run, and 10M generations for the four-model and one-model runs. Finally, we ran Bayesian analyses with BEAST [26] using the same 11 mixed models as the MrBayes analysis under both relaxed and strict clocks.

## Results

### Species relationships

We obtained an unambiguous final alignment of 4,603 bp spanning 11 loci across 62 ingroup and nine outgroup individuals, with only 0.7% gapped or missing data. 223 sites (4.8%) were variable and 108 (2.3%) were parsimony informative. Phylogenetic information content varied by gene, with ZP3 having the greatest proportion of variable sites (8.6%) and GDF9B having the lowest (2.3%; Table 2). Sequences are available from GenBank (Accession numbers PV783005-PV783785).

The three MrBayes analysis schemes generated largely congruent hypotheses, with variability occurring only in the relationship of similar individuals within species or mixed species clades. We therefore only report on the 11-model analysis below.

**Fig 2:**
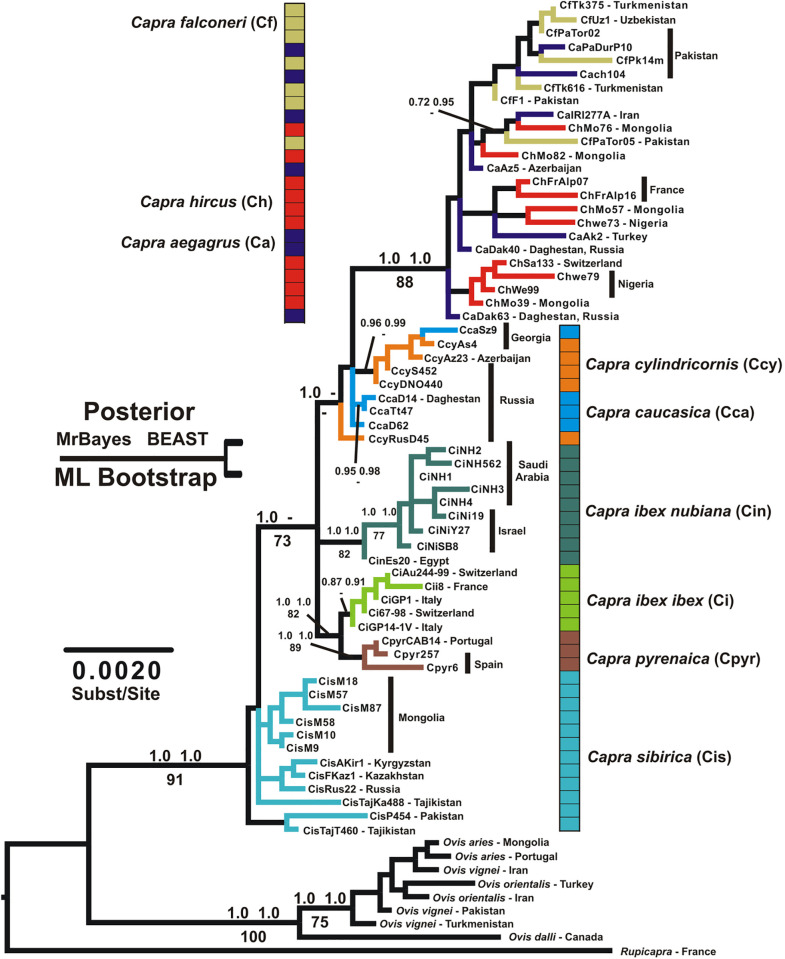
Maximum likelihood phylogram for all taxa generated under the GTR+I+G model using PAUP*. Node labels above branches refer to posterior probabilities generated under the MrBayes “11 model” and the BEAST runs described in the text. Labels below branches refer to bootstrap proportions generated from 175 ML replicates using the GTR+I+G model in PAUP*.

ML and MrBayes analyses also resulted in largely congruent species-level hypotheses. Monophyly of several species was clearly confirmed (Fig 2), including *Capra ibex*, *C. nubiana*, and *C. pyrenaica*. Species of questionable monophyly in one or more analyses include *C. caucasica*, *C. cylindricornis*, *C. falconeri*, *C. aegagrus*, and *C. hircus*.

Monophyly of *C. sibirica* was not precluded by the ML and MrBayes results (Fig 2), and other analyses strongly supported it. These included NJ and BEAST analyses of the entire dataset and NJ analysis of the ZP3 and Kappa Casein loci separately (see S1 File). The results of these analyses were similar to those of MrBayes within nominal species groups, and offered strong support for monophyly of *C. sibirica* (clade posterior = 0.99) and in the placement of deeper branching events (see below).

### Deeper nodes

All analyses found strong support for a large clade consisting of *C. aegagrus*, *C. falconeri,* and *C. hircus* (bezoar, markhor, domestic goat) with unclear relationships between both morphologically defined species and geographic regions. We also found strong support (clade posterior = 1.0) for the sister relationship between *C. ibex* (alpine ibex) and *C. pyrenaica* (Spanish ibex) from southwestern Europe.

**Fig 3:**
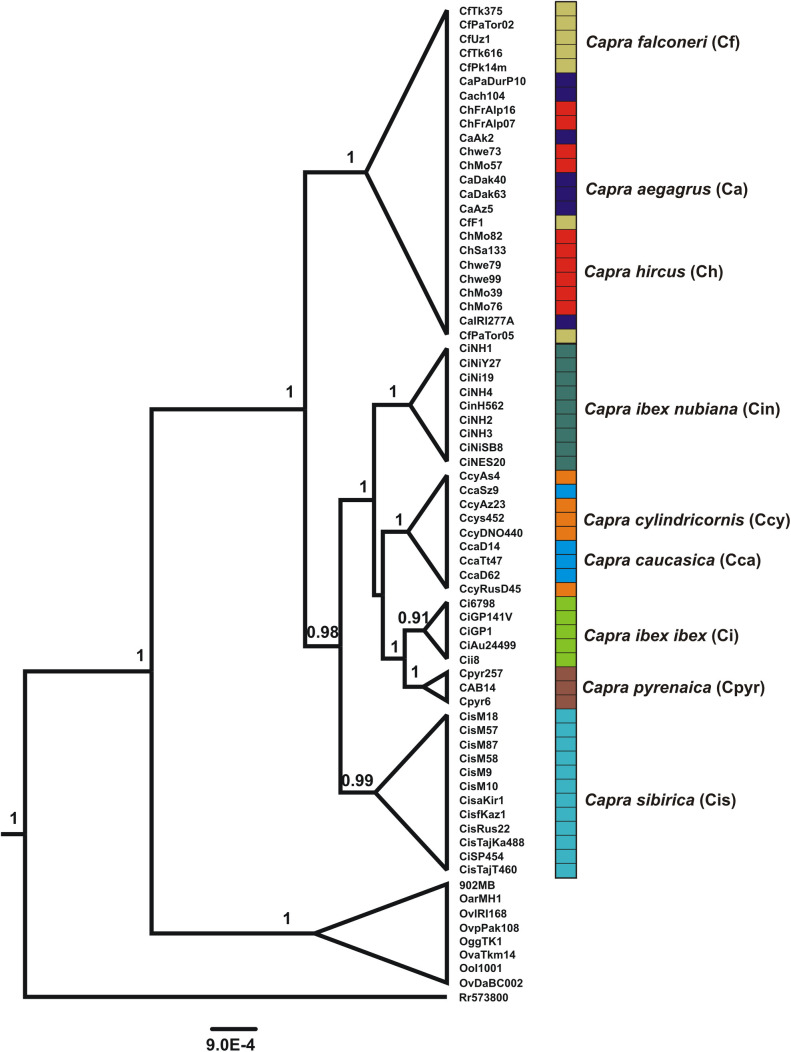
Bayesian topology generated using 11 mixed models and a relaxed clock in BEAST.

The relationship of these groups to all other taxa is not clear in our different analyses. The MrBayes and ML analyses found that the earliest branching events in the genus separated *C. sibirica* from all other species, though with relatively weak ML bootstrap support (73; Fig 2). The BEAST analysis supported a single large clade of ibex-type species, with *C. caucasica* and *C. cylindricornis* (the western and eastern tur) forming a nested monophyletic group without reciprocal monophyly (Fig 3). Therefore, under the BEAST analysis, the initial branching event in the genus was between the goat/bezoar/markhor clade and the ibex/tur clade (Fig 3).

## Discussion

Our *Capra* molecular dataset is notable in terms of taxonomic, genomic, and geographic sampling. It is especially notable for the lack of samples from captive animals. One hallmark of our data is low among-taxa variability within the individual genes (Table 2), which is somewhat surprising given the age of the *Capra* radiation, the widespread geographic distribution of the taxa, and the sequenced loci, which include rapidly evolving reproductive and immune loci. Initially we explored the data using gene-tree/species-tree methods like BEST and STEM [27,28], but the phylogenetic information in each locus was low enough that these analyses were mostly inconclusive. In each of the subsections below, we discuss our results in order, beginning with what is abundantly clear, covering hypotheses that are somewhat supported or not contradicted by our analysis, and ending with more speculative interpretations or suggestions for future research.

### Species relationships

Our analysis clearly confirms several IUCN-recognized species designations, including *C. nubiana*, *C. ibex*, and *C. pyrenaica*, and offers some support for a monophyletic *C. sibirica* (Figs 2, 3). We find evidence for a clade consisting of *C. caucasica* and *C. cylindricornis* (Fig 3), but not for the monophyly of either of these two putative species (Fig 2).

Domestic goats (*C. hircus*) have settled more closely in our analysis with the western bezoar populations than with any other taxon. This is consistent with prior studies hypothesizing that *C. aegagrus* is the most likely ancestor to domestic goats [2,3,7,15,16,29–32].

Although the monophyly of *C. aegagrus*, *C. hircus,* and *C. falconeri* are suspect with respect to each other, perhaps due to natural hybridization (but see [15]), there are nonetheless trends in our data that suggest that currently IUCN-recognized species boundaries of *C. aegagrus* and *C. falconeri* are accurate. For example, a *C. falconeri* from Pakistan found intermingled *C. aegagrus* and *C. hircus* could easily be an artifact of hybridization (Fig 2). Similarly, we also found a clade consisting of all other *C. falconeri*, but that also contains two Pakistani *C. aegagrus* individuals, the easternmost bezoars in our dataset. Future work should include broader genomic coverage and careful gene-by-gene inspection of individual animals to facilitate better understanding of the frequency of hybridization and the locus-specific introgression probabilities in these taxa.

These results are intriguing in light of the findings of Daly et al. [33], who present genomic evidence for a likely extinct tur species whose range and genome both may have overlapped with bezoar-type species and who may have introgressed genetic material into domestic goats. They report that this makes the missing species a possible candidate source for tur-like attributes in *C. hircus*.

Though the ML and MrBayes analyses do not explicitly support monophyly for *C. sibirica*, neither do their nodal support values preclude it (Fig 2). Other analyses support it, including BEAST (Fig 3), with a posterior of 0.99, and NJ analyses of the total dataset, and the ZP3 and kappa casein genes independently. These are the two most variable loci in our dataset, and all other single-gene NJ trees show *C. sibirica* to be scattered around the genus with no clear pattern (S1 File). In other words, when these loci are analyzed individually, there is a lack of signal rather than strong signal for an alternate topology. It is not clear why a monophyletic *C. sibirica* did not emerge from the MrBayes and ML analyses, but these results are intriguing and certainly justify further study of this widespread and likely ancient lineage.

Likewise, although *C. cylindricornis* and *C. caucasica* are clearly very closely related and individually paraphyletic in the ML tree (Fig 2), our analyses hinted at species monophyly, with a core group of *C. cylindricornis* from Russia, Azerbaijan, and Georgia forming a cohesive unit that is only encroached upon by one *C. caucasica* from Georgia (CcaSz9). A single *C. cylindricornis* individual from Russia (CcyRusD45) seems to be quite different from conspecifics as well. Such patterns likely represent real biological events (e.g., hybridization) meriting additional exploration. Future research with additional sampling should evaluate support for formally lumping the current two tur species into a single species.

### Deeper nodes and biogeography

All analyses of our data clearly support monophyly of genus *Capra* and two internal multispecies clades: 1) *C. aegagrus*, *C. falconeri*, and *C. hircus* (the goat clade), and 2) *C. ibex* and *C. pyrenaica*, suggesting a single migration of *Capra* into Europe as previously proposed [9,13,34].

At deeper nodes, two fundamental questions of broad evolutionary patterns in *Capra* remain, with the monophyly of *C. sibirica* being important to both: 1) What are the sister taxa arising from the basal *Capra* node, and 2) Does a large ibex-type clade exist?

Our results are ambiguous on these issues. BEAST analysis supports a single ibex-type clade made up of the turs and all other ibex species, including the Siberian ibex (Fig 3). This clade is sister to the goat (bezoar-type) clade. This is similar to the findings of Pogorevc et al. [17] and Y-chromosome studies [7,35]. Our ML BPs do not explicitly contradict this result, though the single most likely topology we found does (Fig 2).

Our other analyses (MrBayes, ML, and most single-gene NJ runs) support various configurations of *C. sibirica* populations as sister taxa to the rest of the *Capra*, *C. cylindricornis* and *C. caucasica* as sister to the goat (bezoar-type) clade, and non-monophyly of the Siberian ibex (Fig 2 and S1 File). Long branches in the ibex-type group, especially in *C. sibirica*, may be influencing the unsettled behavior of these taxa in our phylogenies.

These results do not necessarily throw into question the hypothesis of Pidancier at al. [7], based on a comparison of nuclear (Y-chromosome) data and mtDNA, that *Capra sibirica* is the most ancient lineage of *Capra* and all others are derived from it. However, a preponderance of nuclear DNA sequence data now supports parallel evolution of ibex-type and bezoar-type clades, with the geographic origin unclear from molecular data.

This study complements recent genomic studies [15–17], and demonstrates the importance of using many loci and individual samples collected from local wild populations (not zoos) from across each species range to assess relationships within and among recently-evolved species and populations. Such work on geographically widespread species, with populations in diverse geopolitical environments, requires collaboration among dozens of scientists, resource managers, rangers, and civic leaders, but can lead to results that validate such intense efforts.

## Supporting information

S1 File Output from exploratory neighbor-joining analyses for all data partitions. This file include the raw output from initial neighbor-joining analyses run in PAUP* for each data partition as well as the entire dataset, and shows initial topology and parameter estimates used to inform subsequent ML and Bayesian analyses.
(PDF)
