# Supplementary material for: A phylogeny for genus Capra based on extensive sampling of wild populations
Source: PLoS One. 2025 Oct 27;20(10):e0334624. doi: 10.1371/journal.pone.0334624 (PMC12558489; doi:10.1371/journal.pone.0334624)
Supplement: S1 File — This file include the raw output from initial neighbor-joining analyses run in PAUP* for each data partition as well as the entire dataset, and shows initial topology and parameter estimates used to inform subsequent ML and Bayesian analyses. (PDF) [file pone.0334624.s001.pdf]

```
P A U P *
Version 4.0a (build 168) for macOS (built on Aug  2 2020 at 06:41:30)
Fri Jun  6 11:49:47 2025


******** Maximum Parsimony (MP) and Neighbor Joining (NJ) analysis of All Data *************

Character-exclusion status changed:
  4307 characters re-included
  Total number of characters now excluded = 0
  Number of included characters = 4603

Heuristic search settings:
  Optimality criterion = parsimony
    Character-status summary:
      Of 4603 total characters:
        All characters are of type 'unord'
        All characters have equal weight
        4381 characters are constant (proportion = 0.951771)
        114 variable characters are parsimony-uninformative
        Number of parsimony-informative characters = 108
    Gaps are treated as "missing"
    Multistate taxa interpreted as uncertainty
  Starting tree(s) obtained via stepwise addition
    Addition sequence: simple (reference taxon = Ca Ak 2)
    Number of trees held at each step = 1
  Branch-swapping algorithm: tree-bisection-reconnection (TBR) with reconnection limit = 8
    Steepest descent option not in effect
  'Maxtrees' setting = 1 (will not be increased)
  Branches collapsed (creating polytomies) if maximum branch length is zero
  'MulTrees' option in effect
  No topological constraints in effect
  Trees are unrooted

Heuristic search completed
  Total number of rearrangements tried = 39100
  Score of best tree(s) found = 323
  Number of trees retained = 1
  Time used = 0.02 sec (CPU time = 0.01 sec)

Tree 1 (rooted using user-specified outgroup)

                                                                          /----------- Ca Ak 2
                                                                          |
                                                                    /-----+    /------ ChMo57
                                                                    |     \----+
                                                   /----------------+          \------ Chwe73
                                                   |                |
                                                   |                |          /------ Ch Fr Alp 07
                                                   |                \----------+
                                             /-----+                           \------ Ch Fr Alp 16
                                             |     |
                                             |     |     /---------------------------- Ca ch 104
                                             |     |     |
                                             |     |     |                     /------ Ca Pa Dur P10
                                             |     \-----+    /----------------+
                                             |           |    |                \------ Cf Pk 14m
                                             |           \----+
                                             |                |     /----------------- Cf Pa Tor 02
                                             |                |     |
                                             |                \-----+     /----------- Cf Tk 375
                                             |                      |     |
                                             |                      \-----+    /------ CfTk616
                                             |                            \----+
                                             |                                 \------ Cf Uk 1
                                             |
                                             |                /----------------------- Ca Az 5
                                             |                |
                                        /----+                |                /------ Ca IRI 277 A
                                        |    |                |           /----+
                                        |    |                |           |    \------ ChMo76
                                        |    |                |     /-----+
                                        |    |                +-----+     \----------- Cf Pa Tor 05
                                        |    |                |     |
                                        |    +----------------+     \----------------- ChMo82
                                        |    |                |
                                        |    |                +----------------------- CfF1
                                        |    |                |
                       /----------------+    |                |           /----------- ChMo39
                       |                |    |                |           |
```

```
                 |                |    |                |     /-----+    /------ Ch Sa 133
                 |                |    |                |     |     \----+
                 |                |    |                \-----+          \------ Chwe79
                 |                |    |                      |
                 |                |    |                      \----------------- Chwe99
                 |                |    |
                 |                |    \---------------------------------------- CaDak40
                 |                |
                 |                \--------------------------------------------- CaDak63
                 |
                 |                                                       /------ CcaD14
                 |                /--------------------------------------+
                 |                |                                      \------ CcaTt47
                 |                |
                 |                +--------------------------------------------- Cca D 62
                 |                |
                 |                |    /---------------------------------------- Ccy Rus D45
                 |                |    |
                 |          /-----+    |                      /----------------- Ci 67 98
                 |          |     |    |                      |
                 |          |     |    |                /-----+          /------ Ci Au244 99
                 |          |     |    |                |     |     /----+
           /-----+          |     |    |                |     |     |    \------ Cii 8
           |     |          |     |    |           /----+     \-----+
           |     |          |     |    |           |    |           \----------- CiGP1
           |     |          |     \----+           |    |
           |     |          |          |           |    \----------------------- Ci GP 14 1V
           |     |          |          |     /-----+
           |     |          |          |     |     |                     /------ Cpyr257
           |     |          |          |     |     |                /----+
           |     |          |          |     |     |                |    \------ CAB 14
           |     |          |          |     |     \----------------+
           |     |          |          |     |                      \----------- C pyr 6
           |     |          |          \-----+
           |     |          |                |     /---------------------------- Ci NES 20
           |     |    /-----+                |     |
           |     |    |     |                |     |          /----------------- CiNH1
           |     |    |     |                |     |          |
           |     |    |     |                |     |          |          /------ CiNH2
           |     |    |     |                |     |          |     /----+
           |     |    |     |                \-----+          |     |    \------ CiNH3
           |     |    |     |                      |          +-----+
           |     |    |     |                      |    /-----+     \----------- CinH562
           |     |    |     |                      |    |     |
           |     |    |     |                      |    |     |          /------ CiNH4
           |     |    |     |                      |    |     +----------+
           |     |    |     |                      \----+     |          \------ CiNi19
           |     |    |     |                           |     |
           |     \----+     |                           |     \----------------- CiNiY 27
           |          |     |                           |
           |          |     |                           \----------------------- CiNiSB 8
     /-----+          |     |
     |     |          |     |                                            /------ CcDNO440
     |     |          |     \--------------------------------------------+
     |     |          |                                                  \------ CcYs452
     |     |          |
     |     |          |                                                  /------ Cca Sz 9
     |     |          |                                                  |
     |     |          \--------------------------------------------------+------ Cc Az 23
     |     |                                                             |
     |     |                                                             \------ Cc As 4
     |     |
     |     |                                                             /------ Cis a Kir 1
     |     |                                                        /----+
     |     |                                                        |    \------ Cis f Kaz 1
     |     +--------------------------------------------------------+
     |     |                                                        \----------- Cis Rus 22
/----+     |
|    |     |                                                             /------ CisM10
|    |     |                                                  /----------+
|    |     |                                                  |          \------ CisM9
|    |     |                                                  |
|    |     |                                                  |          /------ CisM18
|    |     +--------------------------------------------------+          |
|    |     |                                                  |     /----+------ CisM57
|    |     |                                                  |     |    |
|    |     |                                                  \-----+    \------ CisM87
|    |     |                                                        |
|    |     |                                                        \----------- CisM58
|    |     |
|    |     \-------------------------------------------------------------------- Cis Taj Ka488
```

```
      |    |
/-----+    |                                                                   /------ CiSP454
|     |    \-------------------------------------------------------------------+
|     |                                                                        \------ Cis Taj T460
|     |
|     |                                                                        /------ OarMH1
|     |                                                                   /----+
|     |                                                                   |    \------ 902MB
|     |                                                             /-----+
|     |                                                             |     \----------- OvIRI168
|     |                                                       /-----+
|     |                                                       |     +----------------- OggTK1
|     |                                                       |     |
|     |                                                  /----+     \----------------- OvpPak108
|     |                                                  |    |
|     |                                                  |    |                /------ OoI1001
|     \--------------------------------------------------+    \----------------+
|                                                        |                     \------ OvaTkm14
|                                                        |
|                                                        \---------------------------- OvDaBC002
|
\------------------------------------------------------------------------------------- Rr573800a

Neighbor-joining search settings:
  Ties (if encountered) will be broken systematically
  Distance measure = maximum-likelihood
  Likelihood settings:
    Current model:
                             Data type = nucleotide
                DNA substitution types = 6 (restricted to 1 distinct rate)
                     Exchangeabilities = AC=1 AG=1 AT=1 CG=1 CT=1 GT=1
                     State frequencies = empirical: A=0.249886 C=0.260751 G=0.220544 T=0.268819
        Proportion of invariable sites = none
               Rates at variable sites = equal
                  Model correspondence = F81 submodel of GTR

  4603 characters are included
  All characters have equal weight
  (Tree is unrooted)

Neighbor-joining tree:

                                                                        /------------- Ca Ak 2
                                                                        |
                                                                     /--+/--- Ch Fr Alp 07
                                                                     |  \+
                                                                  /--+   \----------- Ch Fr Alp 16
                                                                  |  |
                                                                  |  |   /---------- ChMo57
                                                                  |  \---+
                                                                  |      \----- Chwe73
                                                                 /+
                                                                 || /--- ChMo39
                                                                 || |
                                                                 ||/+ /--- Ch Sa 133
                                                                 |||\-+
                                                                 |\+  \---------- Chwe79
                                                                 | |
                                                                 | \-- Chwe99
                                                                 |
                                                                 +------- ChMo82
                                                                 |
                                                                 +--- Ca Az 5
                                                                 |
                                                                 |  /----------- Ca ch 104
                                                                 |  |
                                                                 |  | /------ Ca Pa Dur P10
                                                                 |  | |
                                                                /+ /+ | /- Cf Pa Tor 02
                                                                || ||/+ |
                                                                || ||||/+ / Cf Tk 375
                                                                || |||||\-+
                                                                || |\+\+  \---- Cf Uk 1
                                                                ||/+ | |
                                                                |||| | \---- CfTk616
                                                                |||| |
                                                                |||| \----------- Cf Pk 14m
                                                              /-+|||
                                                              | |\+\-- CfF1
                                                              | | |
                                                              | | |     /------- Ca IRI 277 A
```

```
                                                              | | |  /--+
                                                              | | |  |  \-------- ChMo76
                                                   /----------+ | \--+
                                                   |          | |    \------------ Cf Pa Tor 05
                                                   |          | |
                                                   |          | \-- CaDak40
                                                   |          |
                                                   |          \--- CaDak63
                                                   |
                                                   |       /-- CcaD14
                                                   |     /-+
                                                   |     | \ CcaTt47
                                                   |    /+
                                                   |    |\--- Cca D 62
                                                   |    |
                                                   |    | /----- Ccy Rus D45
                                                   |    | |
                                                   |    | |          /- Ci 67 98
                                             /-----+   /+ |        /-+
                                             |     |   || |        | \- CiGP1
                                             |     |   || |       /+
                                             |     |   || |       |\- Ci GP 14 1V
                                             |     |   || |      /+
                                             |     |   || |      || / Ci Au244 99
                                             |     |   |\-+      |\-+
                                             |     |   |  |      |  \------ Cii 8
                                             |     |   |  |/-----+
                                             |     |   |  ||     |      /- Cpyr257
                                             |     |   |  ||     |    /-+
                                             |     |   |  ||     |    | \ CAB 14
                                             |     |   |  ||     \----+
                                             |     |   |  ||          \----------- C pyr 6
                                             |     |   |  ||
                                             |     |   |  \+    /---- Ci NES 20
                                             |     |   |   |    |
                                             |     |   |   |    |         / CiNH1
                                             |     |   |   |    |         |
                                             |     \---+   |    |        /+    / CiNH4
                                             |         |   |    |        |\----+
                                             |         |   |    |       /+     \-- CiNi19
                                             |         |   \----+       ||
                                             |         |        |     /-+\--- CiNiSB 8
                                             |         |        |     | |
                                             |         |        |    /+ \- CiNiY 27
                                             |         |        |    ||
                                             |         |        |   /+\----- CinH562
                 /---------------------------+         |        |   ||
                 |                           |         |        \---+\----- CiNH3
                 |                           |         |            |
                 |                           |         |            \-- CiNH2
                 |                           |         |
                 |                           |         |    /---- Cca Sz 9
                 |                           |         |    |
                 |                           |         |    +---- Cc Az 23
                 |                           |         | /--+
                 |                           |         | |  \-- Cc As 4
                 |                           |         \-+
                 |                           |           |  /- CcDNO440
                 |                           |           \--+
                 |                           |              \ CcYs452
                 |                           |
                 |                           |           /----- Cis a Kir 1
                 |                           |         /-+
                 |                           |         | \-- Cis f Kaz 1
                 |                           |      /--+
                 |                           |     /+  \---- Cis Rus 22
                 |                           |     ||
                 |                           |     |\------------- Cis Taj Ka488
                 |                           |  /--+
                 |                           |  |  |    /------------- CiSP454
                 |                           |  |  \----+
                 |                           |  |       \--- Cis Taj T460
/----------------+                           |  |
|                |                           |  |/-- CisM10
|                |                           \--+|
|                |                              ||     /- CisM18
|                |                              ||     |
|                |                              ||  /--+/-- CisM57
|                |                              ||  |  \+
|                |                              \+--+   \----- CisM87
|                |                               |  |
```

```
|                |                               |  \---- CisM58
|                |                               |
|                |                               \ CisM9
|                |
|                |                                             /---- OarMH1
|                |                                            /+
|                |                                            |\-- 902MB
|                |                                            |
|                |                                          /-+  /------------- OggTK1
|                |                                          | |/-+
|                |                                          | || \--- OvpPak108
|                |                                        /-+ \+
|                |                                        | |  \-- OoI1001
|                |                             /----------+ |
|                |                             |          | \------ OvaTkm14
|                |                             |          |
|                \-----------------------------+          \--- OvIRI168
|                                              |
|                                              \---------------------------------- OvDaBC002
|
\------------------------------------------------------------------------------------- Rr573800a

Tree found by neighbor-joining method stored in tree buffer
Time used for tree calculation = 0.00 sec (CPU time = 0.00 sec)

1 tree saved to file "~/Desktop/1a_Capra_2025_Data/NJ_71_2025.tre"

******** Neighbor Joining (NJ) analysis of ZP2x89   *************

Character-exclusion status changed:
  4293 characters excluded
  Total number of characters now excluded = 4293
  Number of included characters = 310

Heuristic search settings:
  Optimality criterion = parsimony
    Character-status summary:
      4293 characters are excluded
      Of the remaining 310 included characters:
        All characters are of type 'unord'
        All characters have equal weight
        295 characters are constant (proportion = 0.951613)
        8 variable characters are parsimony-uninformative
        Number of (included) parsimony-informative characters = 7
    Gaps are treated as "missing"
    Multistate taxa interpreted as uncertainty
  Starting tree(s) obtained via stepwise addition
    Addition sequence: simple (reference taxon = Ca Ak 2)
    Number of trees held at each step = 1
  Branch-swapping algorithm: tree-bisection-reconnection (TBR) with reconnection limit = 8
    Steepest descent option not in effect
  'Maxtrees' setting = 1 (will not be increased)
  Branches collapsed (creating polytomies) if maximum branch length is zero
  'MulTrees' option in effect
  No topological constraints in effect
  Trees are unrooted

Heuristic search completed
  Total number of rearrangements tried = 24860
  Score of best tree(s) found = 18
  Number of trees retained = 1
  Time used = 0.09 sec (CPU time = 0.09 sec)

Neighbor-joining search settings:
  Ties (if encountered) will be broken systematically
  Distance measure = maximum-likelihood
  Likelihood settings:
    Current model:
                             Data type = nucleotide
                DNA substitution types = 6 (restricted to 1 distinct rate)
                     Exchangeabilities = AC=1 AG=1 AT=1 CG=1 CT=1 GT=1
                     State frequencies = empirical: A=0.280719 C=0.21946 G=0.227199 T=0.272622
        Proportion of invariable sites = none
               Rates at variable sites = equal
                  Model correspondence = F81 submodel of GTR

  4293 characters are excluded
  310 characters are included
  All characters have equal weight
  (Tree is unrooted)
```

Neighbor-joining tree:

```
                                                     / Ca Ak 2
                                                     |
                                                     |                   / Ca ch 104
                                                     |                   |
                                                     |                   + CfF1
                                                     |                   |
                                                     +-------------------+ CfTk616
                                                     |                   |
                                                     |                   \ Cf Uk 1
                                                     |
                                                     + Ca Az 5
                                                     |
                                                     + Cf Pa Tor 02
                                                     |
                                                     + Cf Pa Tor 05
                                                     |
                                                     + Cf Pk 14m
                                                     |
                                                     + Cf Tk 375
                                                     |
                                                     + Ch Fr Alp 07
                                                     |
                                                     + Ch Fr Alp 16
                                                     |
                                                     + ChMo39
                                                     |
                                                     + ChMo57
                                                     |
                                                     + ChMo76
                                                     |
                                                     + ChMo82
                                                     |
                                                     + Ch Sa 133
                                                     |
                                                     + Chwe73
                                                     |
                                                     + Chwe79
                                                     |
                                                     + Chwe99
                                                     |
                                                     + CaDak40
                                                     |
                                                     + CaDak63
                                                     |
   /-------------------------------------------------+ Ca IRI 277 A
   |                                                 |
   |                                                 \ Ca Pa Dur P10
   |
   |         / CcaD14
   |         |
   |         |                   / Cc Az 23
   |         |                   |
   |         |                   + CcDNO440
   |         +-------------------+
   |         |                   \ CcYs452
   |         |
   |         + CcaTt47
   |         |
   |         + CisM9
   |         |
   |         + CisM18
   |         |
   |         + Cii 8
   |         |
   |         + CiNH2
   |         |
   |         + CinH562
   |         |
   |         + CiNiY 27
   |         |
   |         +-------------------- Cca D 62
   |         |
   |         + Cca Sz 9
/--+         |
|  |         + Cc As 4
|  |         |
|  |         + Ccy Rus D45
|  |         |
|  |         + CisM10
```

```
            |  |         |
            |  |         + Ci GP 14 1V
            |  |         |
            |  |         + CiNH1
            |  |         |
            |  |         + CiNH4
            |  |         |
            |  |         + CiNiSB 8
            |  |         |
            |  |         + CiNi19
            |  |         |
            |  |         + CiNH3
            |  |         |
            |  |         + Ci NES 20
            |  |         |
            |  |         + CiGP1
            |  |         |
            |  |         + Ci Au244 99
            |  |         |
            |  |         + Ci 67 98
     /------+  |         |
     |      |  |         + CAB 14
     |      |  |         |
     |      |  \---------+ C pyr 6
     |      |            |
     |      |            \ Cpyr257
     |      |
     |      |                 /- Cis a Kir 1
     |      |                /+
     |      |                |\- Cis f Kaz 1
     |      |                |
     |      |                +- CisM57
     |      |                |
     |      |                + CisM58
     |      |                |
     |      |               /+------------------- Cis Taj Ka488
     |      |               ||
     |      |               |\------------------- CisM87
     |      |        /------+
     |      |        |      |                  / Cis Rus 22
/----+      |        |      \------------------+
|    |      |        |                         \ Cis Taj T460
|    |      |        |
|    |      |        |           /..- OarMH1
|    |      |        |           |
|    |      |        |          /+       /........- 902MB
|    |      \--------+          ||  /----+
|    |               |          ||  |    \--------- OggTK1
|    |               |         /+\--+
|    |               |         ||   \....- OvIRI168
|    |               |         ||
|    |               |         |\- OvaTkm14
|    |               |         |
|    |               |        /+ OoI1001
|    |               |        ||
|    |               \--------+\ OvpPak108
|    |                        |
|    |                        \-------------------- OvDaBC002
|    |
|    \----------------------------- CiSP454
|
\---------------------------------------------- Rr573800a

Tree found by neighbor-joining method stored in tree buffer
Note: Ties were encountered; neighbor-joining tree may not be unique
Time used for tree calculation = 0.00 sec (CPU time = 0.00 sec)

1 tree appended to file "~/Desktop/1a_Capra_2025_Data/NJ_71_2025.tre"

******** Neighbor Joining (NJ) analysis of ZP3.X35  *************

Character-exclusion status changed:
  310 characters excluded
  475 characters re-included
  Total number of characters now excluded = 4128
  Number of included characters = 475

Heuristic search settings:
  Optimality criterion = parsimony
    Character-status summary:
      4128 characters are excluded
```

```
      Of the remaining 475 included characters:
        All characters are of type 'unord'
        All characters have equal weight
        435 characters are constant (proportion = 0.915789)
        22 variable characters are parsimony-uninformative
        Number of (included) parsimony-informative characters = 18
    Gaps are treated as "missing"
    Multistate taxa interpreted as uncertainty
  Starting tree(s) obtained via stepwise addition
    Addition sequence: simple (reference taxon = Ca Ak 2)
    Number of trees held at each step = 1
  Branch-swapping algorithm: tree-bisection-reconnection (TBR) with reconnection limit = 8
    Steepest descent option not in effect
  'Maxtrees' setting = 1 (will not be increased)
  Branches collapsed (creating polytomies) if maximum branch length is zero
  'MulTrees' option in effect
  No topological constraints in effect
  Trees are unrooted

Heuristic search completed
  Total number of rearrangements tried = 23764
  Score of best tree(s) found = 46
  Number of trees retained = 1
  Time used = 0.06 sec (CPU time = 0.06 sec)

Neighbor-joining search settings:
  Ties (if encountered) will be broken systematically
  Distance measure = maximum-likelihood
  Likelihood settings:
    Current model:
                             Data type = nucleotide
                DNA substitution types = 6 (restricted to 1 distinct rate)
                     Exchangeabilities = AC=1 AG=1 AT=1 CG=1 CT=1 GT=1
                     State frequencies = empirical: A=0.177746 C=0.316085 G=0.289039 T=0.21713
        Proportion of invariable sites = none
               Rates at variable sites = equal
                  Model correspondence = F81 submodel of GTR

  4128 characters are excluded
  475 characters are included
  All characters have equal weight
  (Tree is unrooted)

Neighbor-joining tree:

                           /------------ Ca Ak 2
                           |
                           + CaDak63
                           |
                           + Cf Pa Tor 05
                           |
                           + Ca Az 5
                           |
                           |           / Ca ch 104
                           +-----------+
                           |           \----------- Cf Pk 14m
                           |
                           + Ca IRI 277 A
                           |
                           + CaDak40
                           |
                           +------------ Ch Fr Alp 07
                           |
                           + Cf Pa Tor 02
                           |
                           +------------ Ch Fr Alp 16
                           |
                           + ChMo82
                           |
                           + ChMo39
                           |
                           +------------ Ch Sa 133
                           |
                           + Chwe73
                           |
                           + Chwe99
                           |
                           + Ca Pa Dur P10
                           |
                           +------------ Cf Uk 1
                           |
```

```
                           + Chwe79
                           |
                           + CfF1
                           |
                           + CfTk616
                   /-------+
                   |       + Cf Tk 375
                   |       |
                   |       |                     / ChMo57
                   |       \---------------------+
                   |                             \ ChMo76
                   |
                   |               /----------- CcaD14
                   |               |
                   |               |/ Cca D 62
                   |               ||
                   |               |+---------------------------------- Cii 8
                   |               ||
                   |               ||                     / Cpyr257
                   |               ||          /----------+
                   |               ||          |          \ CAB 14
                   |               |+----------+
                   |               ||          \----------- C pyr 6
                   |               ||
                   |               |+ CcaTt47
                   |               ||
                   |         /-----++ Ccy Rus D45
                   |         |     ||
                /--+         |     |+ Ci 67 98
                |  |         |     ||
                |  |         |     |+ Ci Au244 99
                |  |         |     ||
                |  |         |     ++ CiGP1
                |  |         |     ||
                |  |         |     |\ Ci GP 14 1V
                |  |         |     |
                |  |         |     |           / Ci NES 20
                |  |         |     |           |
                |  |         |     |           +----------- CiNiSB 8
                |  |         |     |           |
                |  |         |     |           + CiNH1
                |  |     /---+     |           |
                |  |     |   |     |           + CiNH2
                |  |     |   |     |           |
                |  |     |   |     |           + CiNH3
                |  |     |   |     |           |
                |  |     |   |     |           + CiNH4
                |  |     |   |     |           |
                |  |     |   |     |           + CiNi19
                |  |     |   |     |           |
                |  |     |   |     \-----------+ CiNiY 27
                |  \-----+   |                 |
                |        |   |                 \---------------------- CinH562
                |        |   |
                |        |   |     /-------- CcDNO440
     /----------+        |   \-----+
     |          |        |         \--- CcYs452
     |          |        |
     |          |        |                 / Cca Sz 9
     |          |        |                 |
     |          |        |                 + Cc Az 23
     |          |        \-----------------+
     |          |                          \ Cc As 4
     |          |
     |          + Cis a Kir 1
     |          |
     |          |          /- Cis f Kaz 1
     |          +----------+
     |          |          \------------ Cis Taj Ka488
     |          |
     |          + Cis Rus 22
     |          |
     |          + CisM9
     |          |
     |          |           /---------------------- CiSP454
     |          +-----------+
     |          |           \ Cis Taj T460
/----+          |
|    |          + CisM18
|    |          |
|    |          |          / CisM10
```

```
|    |          +-----------+
|    |          |           \ CisM87
|    |          |
|    |          + CisM57
|    |          |
|    |          \ CisM58
|    |
|    |                                                          / OarMH1
|    |                                                          |
|    |                                                          |     /----- OggTK1
|    |                                                          +-----+
|    |                                                          |     \....- OvIRI168
|    |                                                          |
|    |                                                          +----------------------- OvDaBC002
|    |                                                          |
|    |                                                          + OoI1001
|    |                                                          |
|    |                                                          + 902MB
|    |                                                          |
|    \----------------------------------------------------------+ OvaTkm14
|                                                               |
|                                                               \ OvpPak108
|
\------------------------------------------ Rr573800a

Tree found by neighbor-joining method stored in tree buffer
Note: Ties were encountered; neighbor-joining tree may not be unique
Time used for tree calculation = 0.00 sec (CPU time = 0.00 sec)

1 tree appended to file "~/Desktop/1a_Capra_2025_Data/NJ_71_2025.tre"

******** Neighbor Joining (NJ) analysis of GDF9b  *************

Character-exclusion status changed:
  475 characters excluded
  481 characters re-included
  Total number of characters now excluded = 4122
  Number of included characters = 481

Heuristic search settings:
  Optimality criterion = parsimony
    Character-status summary:
      4122 characters are excluded
      Of the remaining 481 included characters:
        All characters are of type 'unord'
        All characters have equal weight
        470 characters are constant (proportion = 0.977131)
        7 variable characters are parsimony-uninformative
        Number of (included) parsimony-informative characters = 4
    Gaps are treated as "missing"
  Starting tree(s) obtained via stepwise addition
    Addition sequence: simple (reference taxon = Ca Ak 2)
    Number of trees held at each step = 1
  Branch-swapping algorithm: tree-bisection-reconnection (TBR) with reconnection limit = 8
    Steepest descent option not in effect
  'Maxtrees' setting = 1 (will not be increased)
  Branches collapsed (creating polytomies) if maximum branch length is zero
  'MulTrees' option in effect
  No topological constraints in effect
  Trees are unrooted

Heuristic search completed
  Total number of rearrangements tried = 25948
  Score of best tree(s) found = 11
  Number of trees retained = 1
  Time used = 0.18 sec (CPU time = 0.18 sec)

Neighbor-joining search settings:
  Ties (if encountered) will be broken systematically
  Distance measure = maximum-likelihood
  Likelihood settings:
    Current model:
                             Data type = nucleotide
                DNA substitution types = 6 (restricted to 1 distinct rate)
                     Exchangeabilities = AC=1 AG=1 AT=1 CG=1 CT=1 GT=1
                     State frequencies = empirical: A=0.23481 C=0.268396 G=0.214752 T=0.282042
        Proportion of invariable sites = none
               Rates at variable sites = equal
                  Model correspondence = F81 submodel of GTR

  4122 characters are excluded
```

```
   481 characters are included
   All characters have equal weight
   (Tree is unrooted)

 Neighbor-joining tree:

                                                   / Ca Ak 2
                                                   |
                                                   +----------------------------------- Cca Sz 9
                                                   |
                                                   + Ca Az 5
                                                   |
                                                   + Ca ch 104
                                                   |
                                                   + CaDak40
                                                   |
                                                   + CaDak63
                                                   |
                                                   + Ca IRI 277 A
                                                   |
                                                   + Ca Pa Dur P10
                                                   |
                                                   + CinH562
                                                   |
                                                   + CcaTt47
                                                   |
                                                   +----------------------------------- Cf Pk 14m
                                                   |
                                                   + Cc Az 23
                                                   |
                                                   + CcDNO440
                                                   |
                                                   + Ccy Rus D45
                                                   |
                                                   + CcYs452
                                                   |
                                                   + Cc As 4
                                                   |
                                                   + CfF1
                                                   |
                                                   + Cf Pa Tor 02
                                                   |
                                                   + Cf Pa Tor 05
                                                   |
                                                   + CcaD14
                                                   |
                                                   + Cf Tk 375
                                                   |
                                                   +----------------------------------- CiNiY 27
                                                   |
                                                   + CfTk616
                                                   |
                                                   + Cf Uk 1
                                                   |
                                                   + Ch Fr Alp 07
                                                   |
                                                   + Ch Fr Alp 16
                                                   |
                                                   + ChMo76
                                                   |
                                                   + Cii 8
                                                   |
                                                   + CiNH3
                                                   |
                                                   + Cca D 62
                                                   |
                                                   + ChMo39
                                                   |
                                                   +----------------------------------- CisM87
                                                   |
                                                   + CisM9
                                                   |
                                                   + CiSP454
                                                   |
                                                   + Cis Rus 22
                                                   |
                                                   + Cpyr257
                                                   |
                                                   + ChMo82
                                                   |
                                                   + Ci NES 20
```

```
                                                   |
                                                   + CiNH4
                                                   |
                                                   |                                 / Ch Sa 133
                                                   |                                 |
                                                   |                                 + Chwe73
                                                   |                                 |
                                                   +---------------------------------+ Chwe79
                                                   |                                 |
                                                   |                                 \ Chwe99
                                                   |
                                                   + CisM10
                                                   |
                                                   + CisM57
                                                   |
                                                   + Cis Taj Ka488
                                                   |
                                                   + C pyr 6
                                                   |
                                                   + Ci 67 98
                                                   |
                                                   + Ci Au244 99
                                                   |
                                                   + Ci GP 14 1V
                                                   |
                                                   + CiNH2
                                                   |
                                                   + CiNiSB 8
                                                   |
                                                   + CiNi19
                                                   |
                                                   + CiNH1
                                                   |
                                                   + CiGP1
                                                   |
                                                   + ChMo57
                                                   |
                                                   + CAB 14
                                                   |
                                                   + Cis Taj T460
                                                   |
                                                   + CisM58
                                                   |
                                                   + CisM18
                                                   |
                 /---------------------------------+ Cis f Kaz 1
                 |                                 |
                 |                                 \ Cis a Kir 1
                 |
                 |                                                                   / OarMH1
                 |                                                                   |
                 |                                                                   + 902MB
                 |                                                                   |
/----------------+                                                                   + OggTK1
|                |                                                                   |
|                |                                                                   + OoI1001
|                |                                                                   |
|                |                                                                   + OvaTkm14
|                |                                                                   |
|                |                                 /---------------------------------+ OvIRI168
|                |                                 |                                 |
|                \---------------------------------+                                 \ OvpPak108
|                                                  |
|                                                  \ OvDaBC002
|
\-------------------------------------------------------------------------------------- Rr573800a

Tree found by neighbor-joining method stored in tree buffer
Note: Ties were encountered; neighbor-joining tree may not be unique
Time used for tree calculation = 0.00 sec (CPU time = 0.00 sec)

1 tree appended to file "~/Desktop/1a_Capra_2025_Data/NJ_71_2025.tre"

********  Neighbor Joining (NJ) analysis of KapCas *************

Character-exclusion status changed:
  481 characters excluded
  493 characters re-included
  Total number of characters now excluded = 4110
  Number of included characters = 493
```

```
Heuristic search settings:
  Optimality criterion = parsimony
    Character-status summary:
      4110 characters are excluded
      Of the remaining 493 included characters:
        All characters are of type 'unord'
        All characters have equal weight
        459 characters are constant (proportion = 0.931034)
        19 variable characters are parsimony-uninformative
        Number of (included) parsimony-informative characters = 15
    Gaps are treated as "missing"
    Multistate taxa interpreted as uncertainty
  Starting tree(s) obtained via stepwise addition
    Addition sequence: simple (reference taxon = Ca Ak 2)
    Number of trees held at each step = 1
  Branch-swapping algorithm: tree-bisection-reconnection (TBR) with reconnection limit = 8
    Steepest descent option not in effect
  'Maxtrees' setting = 1 (will not be increased)
  Branches collapsed (creating polytomies) if maximum branch length is zero
  'MulTrees' option in effect
  No topological constraints in effect
  Trees are unrooted

Heuristic search completed
  Total number of rearrangements tried = 32512
  Score of best tree(s) found = 38
  Number of trees retained = 1
  Time used = 0.08 sec (CPU time = 0.08 sec)

Neighbor-joining search settings:
  Ties (if encountered) will be broken systematically
  Distance measure = maximum-likelihood
  Likelihood settings:
    Current model:
                           Data type = nucleotide
                DNA substitution types = 6 (restricted to 1 distinct rate)
                     Exchangeabilities = AC=1 AG=1 AT=1 CG=1 CT=1 GT=1
                     State frequencies = empirical: A=0.323648 C=0.259537 G=0.167282 T=0.249534
        Proportion of invariable sites = none
               Rates at variable sites = equal
                  Model correspondence = F81 submodel of GTR

  4110 characters are excluded
  493 characters are included
  All characters have equal weight
  (Tree is unrooted)

Neighbor-joining tree:

                                      / Ca Ak 2
                                      |
                                      + Ca Az 5
                                      |
                                      + CaDak40
                                      |
                                      + CaDak63
                                      |
                                      + Ca Pa Dur P10
                                      |
                                      + CcaD14
                                      |
                                      + Cca D 62
                                      |
                                      + Cca Sz 9
                                      |
                                      + CcaTt47
                                      |
                                      + Cc Az 23
                                      |
                                      + CcDNO440
                                      |
                                      + Ccy Rus D45
                                      |
                                      + CcYs452
                                      |
                                      + Cc As 4
                                     /+
                                     |+ CfF1
                                     ||
                                     |+ Cf Pa Tor 02
                                     ||
```

```
                                     |+--------- Cf Pk 14m
                                     ||
                                     |+ Cf Tk 375
                                     ||
                                     |+ CfTk616
                                     ||
                                     |+ Cf Uk 1
                                     ||
                                     |+ Ch Fr Alp 07
                                     ||
                                     |+ Ch Fr Alp 16
                                     ||
                                     |+ Ch Sa 133
                                     ||
                                     |+ Chwe99
                                     ||
                                     ||/- ChMo39
                                     |||
                                     |||      / ChMo57
                                 /---+\+  /---+
                                 |   | |  |   \--------- ChMo82
                                 |   | |/-+
                                 |   | \+ \...- Chwe73
                                 |   |  |
                                 |   |  \.- Chwe79
                     /-----------+   |
                     |           |   +- CiNH3
                     |           |   |
                     |           |   \- CiNH2
                     |           |
                     |           \....- CinH562
                     |
                     |         /--------- Ca IRI 277 A
                     |/--------+
                     ||        \--------- ChMo76
                     ||
                     ||                / Ci 67 98
                     ||                |
                     ||                + Ci Au244 99
                   /-+|                |
                   | ||                + CiGP1
                   | ||                |
                   | ||                + Ci GP 14 1V
                   | ||                |
                   | ||                + Cii 8
                   | ||                |
                   | ||                + Cpyr257
                   | ||                |
                   | |+-----------------+ C pyr 6
                   | ||                |
                   | ||                \ CAB 14
                   | ||
                   | |+------------------ Cf Pa Tor 05
                   | ||
                   | \+       / Ci NES 20
                   |  |       |
      /------------+  |       +--------- CiNi19
      |            |  |       |
      |            |  |       + CiNiSB 8
      |            |  |       |
      |            |  |       + CiNiY 27
      |            |  |       |
      |            |  \-------+ CiNH1
      |            |          |
      |            |          \ CiNH4
      |            |
      |            |    /...- Ca ch 104
      |            |    |
      |            |    |   /--------- Cis a Kir 1
      |            |    |   |
      |            |    |   + Cis f Kaz 1
      |            |    |   |
      |            \----+   +--------- Cis Rus 22
      |                 |   |
      |                 |   + Cis Taj Ka488
      |                 |   |
      |                 |   + Cis Taj T460
      |                 |   |
      |                 \---+ CisM10
      |                     |
/-----+                     + CisM18
```

```
|     |                     |
|     |                     + CisM57
|     |                     |
|     |                     + CisM58
|     |                     |
|     |                     + CisM87
|     |                     |
|     |                     + CisM9
|     |                     |
|     |                     \ CiSP454
|     |
|     |                                     / OarMH1
|     |                                     |
|     |                                     + 902MB
|     |                                     |
|     |                                     + OggTK1
|     |                                     |
|     |                                     + OoI1001
|     |                                     |
|     |                                     + OvaTkm14
|     |                                     |
|     |            /------------------------+ OvIRI168
|     |            |                        |
|     \------------+                        \ OvpPak108
|                  |
|                  \------------------------------------------------------------------ OvDaBC002
|
\--------------------------------------------------------- Rr573800a

Tree found by neighbor-joining method stored in tree buffer
Note: Ties were encountered; neighbor-joining tree may not be unique
Time used for tree calculation = 0.00 sec (CPU time = 0.00 sec)

1 tree appended to file "~/Desktop/1a_Capra_2025_Data/NJ_71_2025.tre"

********  Neighbor Joining (NJ) analysis of Kap1.3  *************

Character-exclusion status changed:
  493 characters excluded
  583 characters re-included
  Total number of characters now excluded = 4020
  Number of included characters = 583

Heuristic search settings:
  Optimality criterion = parsimony
    Character-status summary:
      4020 characters are excluded
      Of the remaining 583 included characters:
        All characters are of type 'unord'
        All characters have equal weight
        560 characters are constant (proportion = 0.960549)
        12 variable characters are parsimony-uninformative
        Number of (included) parsimony-informative characters = 11
    Gaps are treated as "missing"
    Multistate taxa interpreted as uncertainty
  Starting tree(s) obtained via stepwise addition
    Addition sequence: simple (reference taxon = Ca Ak 2)
    Number of trees held at each step = 1
  Branch-swapping algorithm: tree-bisection-reconnection (TBR) with reconnection limit = 8
    Steepest descent option not in effect
  'Maxtrees' setting = 1 (will not be increased)
  Branches collapsed (creating polytomies) if maximum branch length is zero
  'MulTrees' option in effect
  No topological constraints in effect
  Trees are unrooted

Heuristic search completed
  Total number of rearrangements tried = 25861
  Score of best tree(s) found = 31
  Number of trees retained = 1
  Time used = 0.09 sec (CPU time = 0.09 sec)

Neighbor-joining search settings:
  Ties (if encountered) will be broken systematically
  Distance measure = maximum-likelihood
  Likelihood settings:
    Current model:
                             Data type = nucleotide
                DNA substitution types = 6 (restricted to 1 distinct rate)
                     Exchangeabilities = AC=1 AG=1 AT=1 CG=1 CT=1 GT=1
                     State frequencies = empirical: A=0.186613 C=0.360781 G=0.245055 T=0.207551
```

```
        Proportion of invariable sites = none
               Rates at variable sites = equal
                  Model correspondence = F81 submodel of GTR

  4020 characters are excluded
  583 characters are included
  All characters have equal weight
  (Tree is unrooted)

Neighbor-joining tree:

                                 /---------------- Ca Ak 2
                         /-------+
                         |       \------------ Ch Fr Alp 16
                         |
                         |                    / Ca Az 5
                         |                    |
                         |                    +-------------- Ca IRI 277 A
                         |                    |
                         |                    + Ca ch 104
                         |                    |
                         |                    + Ch Sa 133
                         |                    |
                         |                    + ChMo39
                         |                    |
                         |                    +-------------- ChMo57
                         |                    |
                         |                    + ChMo76
                         |                    |
                         |             /------+ ChMo82
                         |             |      |
                         |          /--+      \ Chwe99
                         |          |  |
                  /------+          |  \......- Ch Fr Alp 07
                  |      |          |
                  |      |          |                                  /- Ca Pa Dur P10
                  |      |          |                           /------+
                  |      |          |                           |      \-------------- Cf Pa Tor 05
                  |      |         /+                        /-+
                  |      |         ||              /----------+ \------- Cf Pk 14m
                  |      |         ||              |          |
                  |      |         ||              |          \.....- CfF1
                  |      |         ||            /-+
                  |      |         ||            | |        /----------- Cf Pa Tor 02
                  |      |         ||            | |        |
                  |      |        /+\------------+ \--------+   / Cf Tk 375
                  |      |        ||             |          \---+
                  |      |        ||             |              \ Cf Uk 1
                  |      |        ||             |
                  |      |      /-+|             \ CfTk616
                  |      |      | ||
                  |      |      | |\..- Chwe73
                  |      |      | |
                  |      |      | \- Chwe79
                  |      \------+
                  |             +- CaDak40
                  |             |
                  |             |/ Ci Au244 99
                  |             ||
                  |             |+ Cii 8
                  |             ++
                  |             |\ CiGP1
                  |             |
                  |             \ CaDak63
                  |
                  +- CcaTt47
                  |
                  |             / CiNH1
                  |             |
                  |             + CiNH2
                  |             |
                  |             + CiNH3
                  |             |
                  |             + CiNH4
                  |             |
                  |             + CinH562
                  |             |
                  |             + CiNi19
                  |             |
                  |/------------+ CiNiSB 8
                  ||            |
```

```
                  ++            \ CiNiY 27
                  ||
                  |\ CisM10
                  |
                  +- CisM57
                  |
                  + CisM58
                  |
                  + Cca D 62
                  |
                  +--------------- C pyr 6
                  |
                  + CAB 14
                  |
                  + Cca Sz 9
                  |
                  + Cc Az 23
                  |
                  + Ci GP 14 1V
                  |
                  |       /......- CcDNO440
                  +-------+
                  |       \------- Ccy Rus D45
                  |
                  + Cis f Kaz 1
                  |
                  + Cis Rus 22
                  |
                  + Ci 67 98
                  |
                  + Ci NES 20
                  |
                  + Cc As 4
                  |
                  + Cis Taj T460
                  |
                  + CiSP454
                  |
                  + CisM9
                  |
                  + CisM87
                  |
                  + Cpyr257
                /-+
                | + CcYs452
                | |
                | + CcaD14
             /--+ |
             |  | \ CisM18
   /---------+  |
   |         |  \.- Cis a Kir 1
   |         |
   |         \------------------------------------ Cis Taj Ka488
   |
   |                                  /......- OarMH1
   |                           /------+
   |                           |      \------- 902MB
   |                           |
/--+                   /-------+              / OggTK1
|  |                   |       |   /-----------+
|  |                   |       |   |           \ OoI1001
|  |                /--+       \---+
|  |                |  |           \...- OvpPak108
|  |             /--+  |
|  |             |  |  \----------------------- OvIRI168
|  |             |  |
|  \-------------+  \..- OvaTkm14
|                |
|                \--------------- OvDaBC002
|
\--------------------------- Rr573800a
```

Tree found by neighbor-joining method stored in tree buffer
Note: Ties were encountered; neighbor-joining tree may not be unique
Time used for tree calculation = 0.00 sec (CPU time = 0.00 sec)

1 tree appended to file "~/Desktop/1a_Capra_2025_Data/NJ_71_2025.tre"

********   Neighbor Joining (NJ) analysis of TLR2.X2 *************

Character-exclusion status changed:

```
  583 characters excluded
  468 characters re-included
  Total number of characters now excluded = 4135
  Number of included characters = 468

Heuristic search settings:
  Optimality criterion = parsimony
    Character-status summary:
      4135 characters are excluded
      Of the remaining 468 included characters:
        All characters are of type 'unord'
        All characters have equal weight
        453 characters are constant (proportion = 0.967949)
        8 variable characters are parsimony-uninformative
        Number of (included) parsimony-informative characters = 7
    Gaps are treated as "missing"
    Multistate taxa interpreted as uncertainty
  Starting tree(s) obtained via stepwise addition
    Addition sequence: simple (reference taxon = Ca Ak 2)
    Number of trees held at each step = 1
  Branch-swapping algorithm: tree-bisection-reconnection (TBR) with reconnection limit = 8
    Steepest descent option not in effect
  'Maxtrees' setting = 1 (will not be increased)
  Branches collapsed (creating polytomies) if maximum branch length is zero
  'MulTrees' option in effect
  No topological constraints in effect
  Trees are unrooted

Heuristic search completed
  Total number of rearrangements tried = 32028
  Score of best tree(s) found = 16
  Number of trees retained = 1
  Time used = 0.15 sec (CPU time = 0.15 sec)

Neighbor-joining search settings:
  Ties (if encountered) will be broken systematically
  Distance measure = maximum-likelihood
  Likelihood settings:
    Current model:
                            Data type = nucleotide
                DNA substitution types = 6 (restricted to 1 distinct rate)
                     Exchangeabilities = AC=1 AG=1 AT=1 CG=1 CT=1 GT=1
                     State frequencies = empirical: A=0.287796 C=0.196255 G=0.186447 T=0.329502
        Proportion of invariable sites = none
               Rates at variable sites = equal
                  Model correspondence = F81 submodel of GTR

  4135 characters are excluded
  468 characters are included
  All characters have equal weight
  (Tree is unrooted)

Neighbor-joining tree:

                                   / Ca Ak 2
                                   |
                                   + Ca Az 5
                                   |
                                   |                / Ci NES 20
                                   |                |
                                   |                + CiNH1
                                   |                |
                                   |                + CiNH2
                                   |                |
                                   |                + CiNH3
                                   |                |
                                   |                + CiNH4
                                   |                |
                                   |                + CinH562
                                   |                |
                                   |                + CiNi19
                                   |                |
                                   +----------------+ CiNiSB 8
                                   |                |
                                   |                \ CiNiY 27
                                   |
                                   + CaDak63
                                   |
                                   + Cca D 62
                                   |
                                   + CcDNO440
```

```
                 |
                 + CfF1
                 |
                 + Cf Tk 375
                 |
                 + Ch Fr Alp 16
                 |
                 + ChMo82
                 |
                 + Ci Au244 99
                 |
                 + Ca ch 104
                 |
                 |                / Cpyr257
                 |                |
                 |                + C pyr 6
                 +----------------+
                 |                \ CAB 14
                 |
                 + Ca IRI 277 A
                 |
                 + Cca Sz 9
                 |
                 + Ccy Rus D45
                 |
                 + Cf Pa Tor 02
                 |
                 + CfTk616
                 |
                 + ChMo39
            /----+
            |    + Ch Sa 133
            |    |
            |    + CiGP1
            |    |
            |    + Ci 67 98
            |    |
            |    +----------------- Cis a Kir 1
            |    |
            |    + Cis f Kaz 1
            |    |
            |    + Cis Rus 22
            |    |
            |    + CisM10
            |    |
            |    + CisM18
            |    |
            |    + CisM57
            |    |
            |    + CisM58
            |    |
            |    + CisM87
            |    |
            |    + CisM9
            |    |
            |    + Cii 8
            |    |
            |    + Ca Pa Dur P10
            |    |
            |    + Chwe99
        /---+    |
        |   |    + CcaTt47
        |   |    |
        |   |    + CcYs452
        |   |    |
        |   |    + Cf Pa Tor 05
        |   |    |
        |   |    + Cf Uk 1
        |   |    |
        |   |    + ChMo57
        |   |    |
        |   |    + Chwe73
        |   |    |
        |   |    + Ci GP 14 1V
        |   |    |
        |   |    + Chwe79
        |   |    |
/-------+   |    + ChMo76
|       |   |    |
|       |   |    + Ch Fr Alp 07
|       |   |    |
```

```
                  |       |   |    + Cf Pk 14m
                  |       |   |    |
                  |       |   |    + Cc As 4
                  |       |   |    |
                  |       |   |    + Cc Az 23
                  |       |   |    |
                  |       |   |    + CcaD14
                  |       |   |    |
                  |       |   |    \ CaDak40
                  |       |   |
                  |       |   \....- Cis Taj T460
                  |       |
                  |       \.......- Cis Taj Ka488
                  |
                  |                                          /-------- OarMH1
                  |                                          |
                  |                                     /----+       /- 902MB
                  |                                     |    |       |
                  |                                     |    \-------+               / OggTK1
                  |                               /-----+            \---------------+
/-----------------+                               |     |                            \ OvpPak108
|                 |                               |     |
|                 |                          /----+     \....- OoI1001
|                 |                          |    |
|                 |                          |    | / OvaTkm14
|                 +--------------------------+    \-+
|                 |                          |      \ OvDaBC002
|                 |                          |
|                 |                          \.........- OvIRI168
|                 |
|                 \ CiSP454
|
\------------------------------------------------------------------------------------- Rr573800a

Tree found by neighbor-joining method stored in tree buffer
Note: Ties were encountered; neighbor-joining tree may not be unique
Time used for tree calculation = 0.00 sec (CPU time = 0.00 sec)

1 tree appended to file "~/Desktop/1a_Capra_2025_Data/NJ_71_2025.tre"

********   Neighbor Joining (NJ) analysis of TLR4 *************

Character-exclusion status changed:
  468 characters excluded
  375 characters re-included
  Total number of characters now excluded = 4228
  Number of included characters = 375

Heuristic search settings:
  Optimality criterion = parsimony
    Character-status summary:
      4228 characters are excluded
      Of the remaining 375 included characters:
        All characters are of type 'unord'
        All characters have equal weight
        360 characters are constant (proportion = 0.96)
        7 variable characters are parsimony-uninformative
        Number of (included) parsimony-informative characters = 8
    Gaps are treated as "missing"
    Multistate taxa interpreted as uncertainty
  Starting tree(s) obtained via stepwise addition
    Addition sequence: simple (reference taxon = Ca Ak 2)
    Number of trees held at each step = 1
  Branch-swapping algorithm: tree-bisection-reconnection (TBR) with reconnection limit = 8
    Steepest descent option not in effect
  'Maxtrees' setting = 1 (will not be increased)
  Branches collapsed (creating polytomies) if maximum branch length is zero
  'MulTrees' option in effect
  No topological constraints in effect
  Trees are unrooted

Heuristic search completed
  Total number of rearrangements tried = 27156
  Score of best tree(s) found = 17
  Number of trees retained = 1
  Time used = 0.11 sec (CPU time = 0.11 sec)

Neighbor-joining search settings:
  Ties (if encountered) will be broken systematically
  Distance measure = maximum-likelihood
  Likelihood settings:
```

```
    Current model:
                             Data type = nucleotide
                DNA substitution types = 6 (restricted to 1 distinct rate)
                     Exchangeabilities = AC=1 AG=1 AT=1 CG=1 CT=1 GT=1
                     State frequencies = empirical: A=0.266577 C=0.27845 G=0.170649 T=0.284325
        Proportion of invariable sites = none
               Rates at variable sites = equal
                  Model correspondence = F81 submodel of GTR

  4228 characters are excluded
  375 characters are included
  All characters have equal weight
  (Tree is unrooted)

Neighbor-joining tree:

                                                                     / Ca Ak 2
                                                                     |
                                                    /----------------+ Ch Fr Alp 07
                                                    |                |
                                                    |                + Ch Fr Alp 16
                                   /----------------+                |
                                   |                |                \ ChMo57
                                   |                |
                                   |                \ Chwe73
                                   |
                                   + Ci NES 20
                                   |
                                   + CiNH1
                                   |
                                   + CiNH2
                                   |
                                   + CiNH3
                                   |
                                   + CiNH4
                                   |
                                   + CinH562
                                   |
                                   + CiNi19
                                   |
                                   + CiNiSB 8
                                   |
                                   + CiNiY 27
                                   |
                                   |                / Ca Az 5
                                   |                |
                                   |                +----------------- CisM58
                                   |                |
                                   |                + CaDak40
                                   |                |
                                   |                + CaDak63
                                   |                |
                                   |                + Ca IRI 277 A
                                   |                |
                                   |                + Cis a Kir 1
                                   |                |
                                   |                + Cis f Kaz 1
                                   |                |
                                   |                + CisM10
                                   |                |
                                   |                + CisM18
                                   |                |
                                   |                + CisM57
                                   |                |
                                   |                + CcaD14
                                   |                |
                                   |                + Cca D 62
                                   |                |
                                   |                + Cca Sz 9
                                   |                |
                                   |                + CcaTt47
                                   |                |
                                   |                + Cc Az 23
                                   |                |
                                   |                + Ccy Rus D45
                                   |                |
                                   |                + Cc As 4
                                   |                |
                                   |                + CisM87
                                   |                |
                                   |                + CisM9
                                   |
```

```
                                                   |                |
                                                   |                + CiSP454
                                                   |                |
                                  /----------------+                + Cis Rus 22
                                  |                |                |
                                  |                |                + Cis Taj Ka488
                                  |                |                |
                                  |                |                + Cis Taj T460
                                  |                |                |
                                  |                |                + Cpyr257
                                  |                |                |
                                  |                |                + CAB 14
                                  |                |                |
                                  |                |                + CfF1
                                  |                |                |
                                  |                |                + CcYs452
                                  |                |                |
                                  |                |                + CcDNO440
                                  |                |                |
                                  |                |                + Cii 8
                                  |                |                |
                                  |                |                + Ci GP 14 1V
                                  |                |                |
                                  |                |                + CiGP1
                                  |                |                |
                                  |                |                + Ci Au244 99
                                  |                |                |
                                  |                |                + Ci 67 98
                                  |                |                |
                                  |                |                + Chwe99
                                  |                |                |
                                  |                |                + Chwe79
                                  |                |                |
                                  |                |                + ChMo39
                                  |                |                |
                                  |                |                + ChMo82
                                  |                |                |
                                  |                |                + Ch Sa 133
                                  |                |                |
                                  |                |               /+ ChMo76
                                  |                |               ||
                                  |                |        /------+\ Cf Pa Tor 05
                                  |                |        |      |
                                  |                |        |      \---------------- C pyr 6
                                  |                |        |                    / Ca ch 104
                                  |                |        |                    |
                                  |                \--------+       /------------+ Ca Pa Dur P10
                                  |                         |       |            |
                                  |                         |       |            + Cf Pa Tor 02
                                  |                         |       |            |
                                  |                         |       |            + Cf Tk 375
                                  |                         \-------+            |
/---------------------------------+                                 |            + CfTk616
|                                 |                                 |            |
|                                 |                                 |            \ Cf Uk 1
|                                 |                                 |
|                                 |                                 \---- Cf Pk 14m
|                                 |
|                                 |                / OarMH1
|                                 |        /-------+
|                                 |        |       \ OvIRI168
|                                 +--------+
|                                 |        \.......- 902MB
|                                 |
|                                 |        /.......- OggTK1
|                                 |        |
|                                 +--------+       / OvaTkm14
|                                 |        |       |
|                                 |        |       + OvpPak108
|                                 |        \-------+
|                                 |                \ OvDaBC002
|                                 |
|                                 \ OoI1001
|
\------------------------------------------------------------------------------------- Rr573800a

Tree found by neighbor-joining method stored in tree buffer
Note: Ties were encountered; neighbor-joining tree may not be unique
Time used for tree calculation = 0.00 sec (CPU time = 0.00 sec)
```

```
1 tree appended to file "~/Desktop/1a_Capra_2025_Data/NJ_71_2025.tre"

********   Neighbor Joining (NJ) analysis of U80588 *************

Character-exclusion status changed:
  375 characters excluded
  239 characters re-included
  Total number of characters now excluded = 4364
  Number of included characters = 239

Heuristic search settings:
  Optimality criterion = parsimony
    Character-status summary:
      4364 characters are excluded
      Of the remaining 239 included characters:
        All characters are of type 'unord'
        All characters have equal weight
        225 characters are constant (proportion = 0.941423)
        4 variable characters are parsimony-uninformative
        Number of (included) parsimony-informative characters = 10
    Gaps are treated as "missing"
    Multistate taxa interpreted as uncertainty
  Starting tree(s) obtained via stepwise addition
    Addition sequence: simple (reference taxon = Ca Ak 2)
    Number of trees held at each step = 1
  Branch-swapping algorithm: tree-bisection-reconnection (TBR) with reconnection limit = 8
    Steepest descent option not in effect
  'Maxtrees' setting = 1 (will not be increased)
  Branches collapsed (creating polytomies) if maximum branch length is zero
  'MulTrees' option in effect
  No topological constraints in effect
  Trees are unrooted

Heuristic search completed
  Total number of rearrangements tried = 27072
  Score of best tree(s) found = 19
  Number of trees retained = 1
  Time used = 0.04 sec (CPU time = 0.05 sec)

Neighbor-joining search settings:
  Ties (if encountered) will be broken systematically
  Distance measure = maximum-likelihood
  Likelihood settings:
    Current model:
                             Data type = nucleotide
                DNA substitution types = 6 (restricted to 1 distinct rate)
                     Exchangeabilities = AC=1 AG=1 AT=1 CG=1 CT=1 GT=1
                     State frequencies = empirical: A=0.265342 C=0.180361 G=0.230023 T=0.324274
        Proportion of invariable sites = none
               Rates at variable sites = equal
                  Model correspondence = F81 submodel of GTR

  4364 characters are excluded
  239 characters are included
  All characters have equal weight
  (Tree is unrooted)

Neighbor-joining tree:

                                                                          /-------------------- Ca Ak 2
                                                                          |
                                                                          |                   / Cf Pa Tor 05
                                                                          +-------------------+
                                                                          |                   \ CfTk616
                                                                          |
                                                                          + Chwe99
                                                                          |
                                                                          + Cf Uk 1
                                                                          |
                                                                          + Ch Fr Alp 07
                                                                          |
                                                                          + Cf Pk 14m
                                                                          |
                                                                          + Cf Tk 375
                                                                          |
                                                                          + CfF1
                                                                          |
                                                                          + Cf Pa Tor 02
                                                                          |
                                                                          + Ca IRI 277 A
                                                                          |
```

```
                                                                 + Ca Pa Dur P10
                                                                 |
                                                                 + Ca Az 5
                                                                 |
                                                         /-------+ Ca ch 104
                                                         |       |
                                                         |       \ CaDak40
                                                         |
                                          /--------------+          / ChMo57
                                          |              |          |
                                          |              |          + ChMo76
                                          |              |          |
                                          |              \----------+ ChMo82
                                          |                         |
                         /----------------+                         \ Chwe73
                         |                |
                         |                |   / CaDak63
                         |                |   |
                         |                |   + ChMo39
                         |                \---+
                         |                    |                  /------------------- Ch Fr Alp 16
                         |                    \------------------+
                         |                                       \ Ch Sa 133
                      /--+
                      |  | / Cis a Kir 1
                      |  | |
                      |  | + Cis f Kaz 1
                      |  | |
                      |  | + CiSP454
                      |  | |
                      |  | + Cis Rus 22
                      |  | |
                      |  \-+ Cis Taj Ka488
                      |    |
                      |    \ Cis Taj T460
                      |
                      |                       / CcaD14
                      |                       |
                      |                       +------------------- Chwe79
                      |                       |
                    /-+                       + CiNH1
                    | |                       |
                    | |                       + CcaTt47
                    | |                       |
                    | |                       + CiNH2
                    | |                       |
                    | |                       + CiNH3
                    | |                       |
                    | |                       + CiNH4
                    | |                       |
                    | |                       + CinH562
                    | |                       |
                    | |                       + CiNi19
                    | |                       |
                    | |            /----------+ Cpyr257
                    | |            |          |
                    | |            |          \ CiNiY 27
                    | \------------+
                    |              |       / Cca D 62
                    |              |       |
                    |              |       + Cca Sz 9
                    |              \-------+
                    |                      + Cc Az 23
                    |                      |
                    |                      + CcDNO440
                    |                      |
                    |                      + Ccy Rus D45
                    |                      |
                    |                      + Cc As 4
/-------------------+                      |
|                   |                      + Ci 67 98
|                   |                      |
|                   |                      + CiNiSB 8
|                   |                      |
|                   |                      +-------------------------------------- C pyr 6
|                   |                      |
|                   |                      + Ci Au244 99
|                   |                      |
|                   |                      + CiGP1
|                   |                      |
|                   |                      + Ci GP 14 1V
```

```
 |                   |                      |
 |                   |                      + Ci NES 20
 |                   |                      |
 |                   |                      + Cii 8
 |                   |                      |
 |                   |                      + CAB 14
 |                   |                      |
 |                   |                      \ CcYs452
 |                   |
 |                   |     / CisM10
/+                   |     |
||                   |     + CisM18
||                   |     |
||                   |     + CisM57
||                   |     |
||                   |     + CisM58
||                   |     |
||                   \-----+ CisM87
||                         |
||                         \ CisM9
||
||                                                       /.- OarMH1
||                                                       |
||                                                     /-+ / OggTK1
||                                                     | | |
||                                                     | \-+ OvaTkm14
||                                              /------+   |
||                                              |      |   \ OvpPak108
||                                       /------+      |
||                                       |      |      \.- OvDaBC002
||                                       |      |
||                                    /--+      \......- OvIRI168
||                                    |  |
|\------------------------------------+  \------------- OoI1001
|                                     |
|                                     \..- 902MB
|
\------------ Rr573800a

Tree found by neighbor-joining method stored in tree buffer
Note: Ties were encountered; neighbor-joining tree may not be unique
Time used for tree calculation = 0.00 sec (CPU time = 0.00 sec)

1 tree appended to file "~/Desktop/1a_Capra_2025_Data/NJ_71_2025.tre"

********   Neighbor Joining (NJ) analysis of IL4 *************

Character-exclusion status changed:
  239 characters excluded
  451 characters re-included
  Total number of characters now excluded = 4152
  Number of included characters = 451

Heuristic search settings:
  Optimality criterion = parsimony
    Character-status summary:
      4152 characters are excluded
      Of the remaining 451 included characters:
        All characters are of type 'unord'
        All characters have equal weight
        432 characters are constant (proportion = 0.957871)
        8 variable characters are parsimony-uninformative
        Number of (included) parsimony-informative characters = 11
    Gaps are treated as "missing"
    Multistate taxa interpreted as uncertainty
  Starting tree(s) obtained via stepwise addition
    Addition sequence: simple (reference taxon = Ca Ak 2)
    Number of trees held at each step = 1
  Branch-swapping algorithm: tree-bisection-reconnection (TBR) with reconnection limit = 8
    Steepest descent option not in effect
  'Maxtrees' setting = 1 (will not be increased)
  Branches collapsed (creating polytomies) if maximum branch length is zero
  'MulTrees' option in effect
  No topological constraints in effect
  Trees are unrooted

Heuristic search completed
  Total number of rearrangements tried = 32732
  Score of best tree(s) found = 22
  Number of trees retained = 1
  Time used = 0.16 sec (CPU time = 0.16 sec)
```

```
Neighbor-joining search settings:
  Ties (if encountered) will be broken systematically
  Distance measure = maximum-likelihood
  Likelihood settings:
    Current model:
                            Data type = nucleotide
                DNA substitution types = 6 (restricted to 1 distinct rate)
                     Exchangeabilities = AC=1 AG=1 AT=1 CG=1 CT=1 GT=1
                     State frequencies = empirical: A=0.228034 C=0.229699 G=0.240361 T=0.301906
        Proportion of invariable sites = none
               Rates at variable sites = equal
                  Model correspondence = F81 submodel of GTR

  4152 characters are excluded
  451 characters are included
  All characters have equal weight
  (Tree is unrooted)

Neighbor-joining tree:

                                                                   /- Ca Ak 2
                                                                   |
                                                                   |/----------------- Ca Az 5
                                                                   ||
                                                                   |+ CfF1
                                                                   ||
                                                                   |+ Cf Uk 1
                                                                   ||
                                                                   |+ CcaD14
                                                                   ||
                                                                   |+ Cis a Kir 1
                                                                   ||
                                                                   |+ CiNH1
                                                                   ||
                                                                   |+ CfTk616
                                                                   ||
                                                                   ||                / Ch Fr Alp 07
                                                                   ||                |
                                                                   ||                + Chwe79
                                                                   ||                |
                                                                   ||                + Chwe99
                                                                   ||                |
                                                                   ||       /--------+ Ch Fr Alp 16
                                                                   ||       |        |
                                                                   |+-------+        \ ChMo39
                                                                   ||       |
                                                                   ||       \........- Ch Sa 133
                                                                   ||
                                                                   |+ Chwe73
                                                                   ||
                                                                   |+ CiNiY 27
                                                                   ||
                                                                   |+ Ci NES 20
                                                                   ||
                                                                   ||               / CaDak40
                                                                   ||       /-------+
                                                                   ||       |       \ Ca IRI 277 A
                                                                   ||   /---+
                                                                   |+---+   \--------- Ca Pa Dur P10
                                                                   ||   |
                                                                   ||   \...- Cf Pa Tor 05
                                                                   ||
                                                                   |+ Cf Pk 14m
                                                                   ||
                                                                   |+ ChMo57
                                                                   ||
                                                                   |+ Ci GP 14 1V
                                                                   ||
                                                                   |+ Cis f Kaz 1
                                                                   ||
                                                                   |+ CiNH2
                                                                   ||
                                                                   |+ C pyr 6
                                                                   ||
                                                                   |+ Cc Az 23
                                                                   ||
                                                                   |+ CcYs452
                                                                   ||
                                                                   |+ Cf Pa Tor 02
                                                                   ||
```

```
                                                                   |+ ChMo76
                                                                   ||
                                                                   |+ Cf Tk 375
                                                                   ||
                                                                   |+ ChMo82
                                                                   ||
                                                                   |+ CiNiSB 8
                                                                   ||
                                                                   |+ CinH562
                                                                   ||
                                                                   |+ Cpyr257
                                                                   ||
                                                                   |+ Cis Taj T460
                                                                   ||
                                                                   |+ Cis Taj Ka488
                                                                   ||
                                                                   |+ Cis Rus 22
                                                                  /+|
                                                                  |||               / Cca Sz 9
                                                                  |||               |
                                                                  |||               + Ccy Rus D45
                                                                  |||               |
                                                                  |||               + CiSP454
                                                                  |||               |
                                                                  |||               + Cii 8
                                                                  |||               |
                                                                  |||               + Ci 67 98
                                                                  |||               |
                                                                  |||       /-------+ Ci Au244 99
                                                                  |||       |       |
                                                                  |||   /---+       \ CiGP1
                                                                  |||   |   |
                                                                 /+||/--+   \.......- CisM10
                                                                 |||||  |
                                                                 ||++|  \...- CisM9
                                                                 |||||
                                                                 |||++               / CisM18
                                                                 |||||               |
                                                                 |||||               + CisM57
                                                                 |||||      /--------+
                                                                 ||||\------+        \ CisM58
                                                                 ||||       |
                                                                 ||||       \........- CisM87
                                                                 ||||
                                                                 |||\ Ca ch 104
                                                                 |||
                                                                 ||\- CAB 14
                                                                 ||
                                                                 |\.- CcaTt47
                                                   /-------------+
                                                   |             |     /......- CaDak63
                                                   |             | /---+
                                                   |             | |   \------- Cc As 4
                                                   |             +-+
                                                   |             | \...- CcDNO440
    /----------------------------------------------+             |
    |                                              |             \.- Cca D 62
    |                                              |
    |                                              |     / CiNH3
    |                                              |     |
    |                                              |     + CiNH4
    |                                              \-----------------+
    |                                                    \ CiNi19
    |
/---+                      / OarMH1
|   |             /--------+
|   |             |        \ 902MB
|   |    /--------+
|   |    |        \........- OoI1001
|   |    |
|   |    + OvpPak108
|   |    |
|   |    + OvIRI168
|   \----+
|        |       /-------------------------- OggTK1
|        |  /----+
|        |  |    \.......- OvDaBC002
|        \--+
|           \------------- OvaTkm14
|
\------------------------------------------------------------------------------------- Rr573800a
```

```
Tree found by neighbor-joining method stored in tree buffer
Note: Ties were encountered; neighbor-joining tree may not be unique
Time used for tree calculation = 0.00 sec (CPU time = 0.00 sec)

1 tree appended to file "~/Desktop/1a_Capra_2025_Data/NJ_71_2025.tre"

********   Neighbor Joining (NJ) analysis of IL16 *************

Character-exclusion status changed:
  451 characters excluded
  432 characters re-included
  Total number of characters now excluded = 4171
  Number of included characters = 432

Heuristic search settings:
  Optimality criterion = parsimony
    Character-status summary:
      4171 characters are excluded
      Of the remaining 432 included characters:
        All characters are of type 'unord'
        All characters have equal weight
        406 characters are constant (proportion = 0.939815)
        16 variable characters are parsimony-uninformative
        Number of (included) parsimony-informative characters = 10
    Gaps are treated as "missing"
    Multistate taxa interpreted as uncertainty
  Starting tree(s) obtained via stepwise addition
    Addition sequence: simple (reference taxon = Ca Ak 2)
    Number of trees held at each step = 1
  Branch-swapping algorithm: tree-bisection-reconnection (TBR) with reconnection limit = 8
    Steepest descent option not in effect
  'Maxtrees' setting = 1 (will not be increased)
  Branches collapsed (creating polytomies) if maximum branch length is zero
  'MulTrees' option in effect
  No topological constraints in effect
  Trees are unrooted

Heuristic search completed
  Total number of rearrangements tried = 30909
  Score of best tree(s) found = 26
  Number of trees retained = 1
  Time used = 0.12 sec (CPU time = 0.12 sec)

Neighbor-joining search settings:
  Ties (if encountered) will be broken systematically
  Distance measure = maximum-likelihood
  Likelihood settings:
    Current model:
                             Data type = nucleotide
                DNA substitution types = 6 (restricted to 1 distinct rate)
                     Exchangeabilities = AC=1 AG=1 AT=1 CG=1 CT=1 GT=1
                     State frequencies = empirical: A=0.231506 C=0.297542 G=0.264563 T=0.20639
        Proportion of invariable sites = none
               Rates at variable sites = equal
                  Model correspondence = F81 submodel of GTR

  4171 characters are excluded
  432 characters are included
  All characters have equal weight
  (Tree is unrooted)

Neighbor-joining tree:

                                                      / Ca Ak 2
                                                      |
                                                      |                               / Cis a Kir 1
                                                      |                               |
                                                      |                               + Cis Rus 22
                                                      +-------------------------------+
                                                      |                               \ Cis f Kaz 1
                                                      |
                                                      + Cpyr257
                                                      |
                                                      + CisM58
                                                      |
                                                      |              / Ca Az 5
                                                      |              |
                                                      |              +---------------- Ca ch 104
                                                      |              |
                                                      |              + Chwe99
```

```
|               |
|               + CfF1
|               |
|               + Cf Pk 14m
|               |
|               + Cf Uk 1
|               |
|               + ChMo39
|               |
|               + Ch Sa 133
|               |
|               + CaDak40
|               |
|               +---------------- CaDak63
|               |
|               + CcDNO440
|               |
|               + Cf Pa Tor 02
|               |
|               + Cf Tk 375
|               |
|               + Ch Fr Alp 07
|               |
|               + ChMo76
|               |
|               + Ca IRI 277 A
|               |
|               +---------------- Cc Az 23
|               |
|               + Ca Pa Dur P10
|               |
|               + CcaD14
|               |
|               + Cca Sz 9
|               |
|               + CcaTt47
|               |
|               + ChMo57
|               |
|               + ChMo82
|               |
|               + Ch Fr Alp 16
|               |
|               + CfTk616
|               |
|               + Cf Pa Tor 05
|               |
|               + Cc As 4
|               |
+---------------+ Cca D 62
|               |
|               \ CcYs452
|
+ CAB 14
|
+ CisM9
|
+ CisM87
|
+ CiNiY 27
|
+ CiNH4
|
+ CiNH3
|
+ Ci NES 20
|
+ CisM18
|
+ CisM10
|
+ CiNiSB 8
|
+ CiNi19
|
+ CinH562
|
+ CiNH2
|
+ Ccy Rus D45
|
```

```
                                                     |              / Chwe73
                                                     +--------------+
                                                     |              \ Chwe79
                                                     |
                                                     + CiNH1
                                                     |
                                                     + C pyr 6
                                                     |
                                                     |              / Ci 67 98
                                                     |              |
                                                     +--------------+ CiGP1
                                                     |              |
                                                     |              \ Ci GP 14 1V
                                                     |
                                                     |                      /-------- CiSP454
                                                     |   /------------------+
                                                     |   |                  \-------- Cis Taj T460
                                                     +---+
                                                     |   \...- Cis Taj Ka488
                                                     |
                       /-----------------------------+ Cii 8
                       |                             |
                       |                             + Ci Au244 99
                       |                             |
                       |                             \ CisM57
                       |
                       |                       /......- OarMH1
/----------------------+               /-------+
|                      |               |       \------- OggTK1
|                      |               |
|                      |               + OoI1001
|                      |               |
|                      |    /----------+ OvaTkm14
|                      |    |          |
|                      |    |          \ OvIRI168
|                      \----+
|                           |           /..............- 902MB
|                           |   /-------+
|                           |   |       \------------------------------- OvDaBC002
|                           \---+
|                               \------- OvpPak108
|
\---------------------------------------------------------------------------------- Rr573800a

Tree found by neighbor-joining method stored in tree buffer
Note: Ties were encountered; neighbor-joining tree may not be unique
Time used for tree calculation = 0.00 sec (CPU time = 0.00 sec)

1 tree appended to file "~/Desktop/1a_Capra_2025_Data/NJ_71_2025.tre"

********   Neighbor Joining (NJ) analysis of CWC15 *************

Character-exclusion status changed:
  432 characters excluded
  296 characters re-included
  Total number of characters now excluded = 4307
  Number of included characters = 296

Heuristic search settings:
  Optimality criterion = parsimony
    Character-status summary:
      4307 characters are excluded
      Of the remaining 296 included characters:
        All characters are of type 'unord'
        All characters have equal weight
        286 characters are constant (proportion = 0.966216)
        3 variable characters are parsimony-uninformative
        Number of (included) parsimony-informative characters = 7
    Gaps are treated as "missing"
    Multistate taxa interpreted as uncertainty
  Starting tree(s) obtained via stepwise addition
    Addition sequence: simple (reference taxon = Ca Ak 2)
    Number of trees held at each step = 1
  Branch-swapping algorithm: tree-bisection-reconnection (TBR) with reconnection limit = 8
    Steepest descent option not in effect
  'Maxtrees' setting = 1 (will not be increased)
  Branches collapsed (creating polytomies) if maximum branch length is zero
  'MulTrees' option in effect
  No topological constraints in effect
  Trees are unrooted
```

```
Heuristic search completed
  Total number of rearrangements tried = 32172
  Score of best tree(s) found = 11
  Number of trees retained = 1
  Time used = 0.16 sec (CPU time = 0.15 sec)

Neighbor-joining search settings:
  Ties (if encountered) will be broken systematically
  Distance measure = maximum-likelihood
  Likelihood settings:
    Current model:
                             Data type = nucleotide
                DNA substitution types = 6 (restricted to 1 distinct rate)
                     Exchangeabilities = AC=1 AG=1 AT=1 CG=1 CT=1 GT=1
                     State frequencies = empirical: A=0.32286 C=0.149995 G=0.170883 T=0.356262
        Proportion of invariable sites = none
               Rates at variable sites = equal
                  Model correspondence = F81 submodel of GTR

  4307 characters are excluded
  296 characters are included
  All characters have equal weight
  (Tree is unrooted)

Neighbor-joining tree:

/ Ca Ak 2
|
|                                          / Cis a Kir 1
|                                          |
|                                         /+                                         / CisM18
|                                         ||                                         |
|                                         ||                                         + CisM87
|                                         |\-----------------------------------------+
|                                         |                                          \ CisM57
|                                         |
|                                         +- Cis f Kaz 1
|                                         |
+-----------------------------------------+ CiSP454
|                                         |
|                                         \ Cis Taj Ka488
|
+ Ca ch 104
|
+ Cca Sz 9
|
+ Ccy Rus D45
|
+ Cf Pa Tor 02
|
+ CcaTt47
|
+------------------------------------------ 902MB
|
+ OggTK1
|
+ OvpPak108
|
+ CcYs452
|
+ Cf Tk 375
|
+ Cf Uk 1
|
|                                         / ChMo39
|                                         |
|                                         + ChMo57
|                    /--------------------+
+--------------------+                    \ Chwe73
|                    |
|                    \....................- Ch Sa 133
|
+ CAB 14
|
+ Ci 67 98
|
+ CiGP1
|
+ Cii 8
|
|    /.....- CaDak40
```

```
|    |
|    |                                   / Ca IRI 277 A
|    |                                   |
|    |                                   + Cf Pa Tor 05
|    |                                   |
|    |                                   + Ch Fr Alp 07
|    |                                   |
|    |                                   + Ch Fr Alp 16
|  /-+                                   |
|  | |                                   + ChMo76
|  | |                /------------------+
|  | |                |                  + ChMo82
|  | |                |                  |
|  | |                |                  + Cf Pk 14m
+--+ |      /---------+                  |
|  | |      |         |                  \ Ca Pa Dur P10
|  | \------+         |
|  |        |         \..................- Chwe79
|  |        |
|  |        \.........- Chwe99
|  |
|  \.- CaDak63
|
+ Ci Au244 99
|
+ Ci GP 14 1V
|
+ Ci NES 20
|
|                                                                                  / CiNH1
|                                                                                  |
|                                                                                  + CiNiSB 8
|                                                                                  |
|                                                                                  + CiNH4
|                                                                                  |
|                                         /----------------------------------------+ CinH562
|                                         |                                        |
|                               /---------+                                        \ CiNi19
|                               |         |
|               /---------------+         \........................................- CiNiY 27
|               |               |
+---------------+               \----------- CiNH3
|               |
|               \-------------------------- CiNH2
|
+ Cis Taj T460
|
+ C pyr 6
|
+ Cis Rus 22
|
+ CisM10
|
+ CisM58
|
+ CisM9
|
+ Cca D 62
|
|                    /....................- OarMH1
|                    |
+--------------------+                    / OoI1001
|                    \--------------------+
|                                         \ OvaTkm14
|
+ OvIRI168
|
+ CcDNO440
|
+ CfF1
|
+ CfTk616
|
+ Cc As 4
|
+ Cc Az 23
|
+ CcaD14
|
+ Ca Az 5
|
```

```
+ Cpyr257
|
+ OvDaBC002
|
\------------------------------------------------------------------------------------- Rr573800a

Tree found by neighbor-joining method stored in tree buffer
Note: Ties were encountered; neighbor-joining tree may not be unique
Time used for tree calculation = 0.00 sec (CPU time = 0.00 sec)

1 tree appended to file "~/Desktop/1a_Capra_2025_Data/NJ_71_2025.tre"

Processing of input file "GeneDesc2025.nex" completed.
```
